# An empirical assessment of baseline feature location techniques

**DOI:** 10.1007/s10664-019-09734-5

**Published:** 2019-07-16

**Authors:** Abdul Razzaq, Andrew Le Gear, Chris Exton, Jim Buckley

**Affiliations:** 1 Lero, CSIS Department, University of Limerick, Limerick, Ireland; 2 Horizon Globex Ireland DAC, Limerick, Ireland

**Keywords:** Systematic literature review, Feature location, Information retrieval, Concept location

## Abstract

Feature Location (FL) aims to locate observable functionalities in source code. Considering its key role in software maintenance, a vast array of automated and semi-automated Feature Location Techniques (FLTs) have been proposed. To compare FLTs, an open, standard set of non-subjective, reproducible “compare-to” FLT techniques (baseline techniques) should be used for evaluation. In order to relate the performance of FLTs compared against different baseline techniques, these compare-to techniques should be evaluated against each other. But evaluation across FLTs is confounded by empirical designs that incorporate different FL goals and evaluation criteria. This paper moves towards standardizing FLT comparability by assessing eight baseline techniques in an empirical design that addresses these confounding factors. These baseline techniques are assessed in twelve case studies to rank their performance. Results of the case studies suggest that different baseline techniques perform differently and that VSM-Lucene and LSI-Matlab performed better than other implementations. By presenting the relative performances of baseline techniques this paper facilitates empirical cross-comparison of existing and future FLTs. Finally, the results suggest that the performance of FLTs partially depends on system/benchmark characteristics, in addition to the FLTs themselves.

## Introduction

A feature is an observable functionality in a software system that can be triggered by the user (Eisenbarth et al. 2003). Feature Location (FL) concerns itself with the location of feature-related, source code elements. Since the 2000s, hundreds of articles to address the task of feature location have been published in software engineering venues (Razzaq et al. 2018; Dit et al. 2013; Rubin and Chechik 2013; Cornelissen et al. 2009). Notably influential works include Chen and Rajlich (2000), whose technique achieved FL through the examination of the software’s structure via a dependency graph, Wilde et al. (2001), who used program traces gathered during dynamic analysis, and Antoniol et al. (2002), who used an Information Retrieval (IR) technique (textual analysis) to support the feature location task. From these early efforts, the number of structural and textual analysis approaches for FL has expanded dramatically and many new FLTs have been developed (Chen and Rajlich 2000; Antoniol et al. 2002; Lukins et al. 2008; Marcus and Maletic 2003; Marcus et al. 2004; Starke et al. 2009) tailored to different software maintenance activities (Cornelissen et al. 2009). Also, original FLTs have been gradually refined with the intention of enhancing their efficacy. Scanniello et al. (2015), Saha et al. (2013), Revelle et al. (2011), and Rao and Kak (2011), Poshyvanyk et al.(2006, 2012), Panichella et al. (2013), Mahmoud and Bradshaw (2015), Kagdi et al. (2013), Heck and Zaidman (2014), Cleary et al. (2009), Chochlov et al. (2017), Binkley et al. (2015), Bassett and Kraft (2013), and Ali et al. (2013).

However, this proliferation of FLTs means it can be difficult to compare across these techniques (Razzaq et al. 2018). Comparison to commonly used baseline techniques would facilitate cross-comparison and ultimately, may enable developers to choose an appropriate FL technique for a given software maintenance task. In addition, it would also allow researchers to identify the state-of-the-art FL techniques on which to build newly proposed FL solutions. A baseline technique in this context is defined as the one which:

1. Has a track record as a comparison technique.
2. Is openly-available and reproducible where researchers can repeatedly apply it in comparison to novel FLTs being developed.
3. Is original - i.e. Not a refined version of an existing technique or a hybrid.
4. Has a fully defined, complete solution to the FL problem including pre-processing and post-processing steps embedded.
5. Is objective and programmer-independent such that it does not require any intelligent assistance from the programmer in the FLT process, as this would lead to inconsistent results when applied by different users.

The baseline techniques are all IR based. This is not a deliberate bias toward IR, but instead the natural outcome of choosing techniques adhering to the above criteria (Razzaq et al. 2018). To date only a limited number of FLTs have been evaluated against baseline techniques (Gethers et al. 2011; Wang et al. 2011; Dit et al. 2015; Mahmoud and Niu 2015; Marcus and Maletic 2003) and the empirical designs employed have not always been cognizant of confounding factors which may affect they outcome of the evaluations, like the user’s goal and the different evaluation measures employed.

This paper addresses these issues to provide a stronger baseline FLT foundation for FLT researchers. It performs a comparison across baseline techniques to assess which are the better performers and how they rank with respect to each other. In addition, it formally defines empirical designs that address many of the confounding factors in empirical studies of FLTs in the literature. This will allow more accurate comparison of FLTs in the future. Hence we focus on the following four research questions:

1. Do the different implementations of identically-named baseline techniques perform exactly the same? If different implementations of the original baseline techniques perform similarly then it enables as-is cross-comparison of the existing body of FLTs compared to any implementation of the identically named baseline techniques;
2. If not, what are the outstanding implementations of the identically-named baseline techniques for specific empirical designs, defined by FL goals and evaluation measures? That is, if different implementations of the identically-named baseline techniques perform differently, which implementation is better than the others in each case study? This identifies the best performing implementation of each, to be used as a comparator for newly proposed techniques;
3. How do all the baseline techniques compare to each other for specific empirical designs defined on the basis of FL goals and measures? This will facilitate comparison across FLTs that have not been compared to the same baseline technique by relating the performance of baseline techniques with each other. It also allows the indirect comparison of the FLTs that are not compared with any other technique, when evaluated using a data-set which a baseline technique has been/can be evaluated against. In addition, this will identify the best performing baseline technique overall, to be used as a comparator, when studies compare a newly proposed technique to a single baseline technique (as is customary in the field).
4. Based on the results obtained in this study, it seemed that the performance of the FLTs depended on the intrinsic characteristics of the systems/benchmarks they were applied to. We assessed if this was so: If differences in systems impact on FLTs performance, then, the selection of systems to evaluate FLTs should be cognisant of the system’ characteristics that impact on performance.

We show that different implementations of baseline techniques perform differently and thus present approximate rating factors, that could be used in comparisons against different baseline FLTs. This facilitates comparison across the existing body of FLTs that have been compared against baseline techniques. We also identify a best-performing baseline implementation that should be used as a comparator for newly proposed techniques in the field. In addition to these, our findings prompt research towards system and benchmark selection for FLTs evaluation. Finally, we assist researchers towards reproducible and comparable empirical design, cognizant of FL goals and evaluation criteria, with an illustrative example of our empirical design. In this vein, we provide case study data, intermediate results, and implementations online^1^ for transparency and to encourage others to base their design on, replicate and extend our work.

The remainder of this paper is organized as follows: Section 2 reviews background and related work. Section 3 presents the empirical design, particularly with respect to FL goals and the evaluation criteria, used to assess the baseline techniques. The results obtained after employing the empirical design are analyzed for each of the four research questions and are presented in section 4. The paper discusses key findings and how to use the relative baseline performance to compare the existing body of FLTs, in Section 4.5. Section 6 lists the potential threats to the validity of our results. Finally, we conclude and outline future work in Section 7.

## Background and Related Work

A feature is an observable system requirement, functionality or behaviour that can be triggered by users (Eisenbarth et al. 2003). A software system has a set of features where each feature is implemented through a set of source code elements known as the “extent of that feature,” as formally defined by Revelle et al. (2011). The concern of Feature location (FL) is to identify source code elements implementing a feature. This task is intrinsically associated with software maintenance and evolution activities which frequently mandate the location of a feature’s code to document, configure, add, remove, or improve on some functionality (Poshyvanyk et al. 2007).

### Classification of FLTs

A distinguishing factor of FLTs is the type of analyses performed. The most common types of analysis in FLTs are textual, structural, historical and dynamic (Dit et al. 2013). Textual analysis attempts to exploit the domain knowledge already encoded by the developers in the form of comments and identifier-names in a program. The analysis relies on some sort of textual user-query and matching it against these comments or identifiers. Natural Language Processing (NLP), Information Retrieval (IR) and Pattern Matching (PM) are the main analysis techniques employed in textual analysis (Binkley et al. 2015; Diaz et al. 2013; Liu et al. 2007) with the emphasis on IR, as it is more effective than PM while being less complex than NLP (Wang et al. 2011).

Structural analysis, often referred to as static analysis, allows developers to identify the relevant program elements by following data or control flow dependencies between them. For example, if one procedure (or method) is known to be part of the feature and it is the only caller of another procedure, then it is considered likely that this latter procedure is also part of the feature (Bassett and Kraft 2013; Scanniello et al. 2015).

With historical analysis, artifacts related to the feature are identified by mining change-histories available in online source code repositories. For example, if a procedure is known to be in a feature, procedures that tend to change in the same commits as that procedure might also be likely candidate locations for that feature (Chochlov et al. 2017; Wang and Lo 2014; Ye et al. 2016).

Finally, dynamic analysis refers to the invocation and observation of features at execution time: Execution traces are analyzed to identify code that is always executed when the feature is exercised in the system, and code that is not executed when the feature is not, thus identifying code that is (exclusively) associated with the feature (Poshyvanyk et al. 2007; Liu et al. 2007).

More recently, approaches that leverage some combination of these types of analysis have been proposed. These are referred to as ‘hybrids’ (Dit et al. 2013). The purpose of this hybridization is to compensate for the limitations of each individual FLT type, and thus to achieve better overall results than either would perform individually.

### Information Retrieval Process and Models in Feature Location

The FL research problem is mostly interpreted as an IR problem (Antoniol et al.^2002^; Marcus and Maletic 2003; Marcus et al. 2004; Ali et al. 2013; Binkley et al.^2015^; Chochlov et al. 2017; Cleary et al. 2009; Heck and Zaidman 2014; Revelle et al. 2011; Rao and Kak 2011; Poshyvanyk et al. 2007, 2006, 2012; Mahmoud and Niu 2015; Wang et al. 2011; Zhou et al. 2012; Wang et al. 2014; De Lucia et al. 2011; Lukins et al. 2010; Mills et al. 2017; Thomas et al.^2013^; Biggers et al. 2014; Hill et al. 2015; Eaddy et al. 2008; Dit et al.^2011^; Beard et al. 2011). Hence, existing FLTs are predominantly IR-based FLTs. For example, 89% of the FLTs (151 out of 170) reported by Razzaq et al. (2018) and half of the FLTs reported by Dit et al. (2013) required a textual input to retrieve relevant features. In this section, we introduce the IR-based process of feature location embedded in most textual techniques and describe three generic models underpinning them (referred to here as “IR models”):

- Vector Space Models (VSM), an algebraic model;
- Latent Semantic Indexing (LSI), a semantic topic model;
- Latent Dirichlet Allocation (LDA), a probabilistic topic model.

#### The Inherent IR-based, Feature Location Process

Information retrieval is the activity of tracing and recovering relevant information from a collection of documents, given an information need. In FL, an information retrieval model provides a means of identifying source code elements from source code “documents” likely to contribute to the implementation of a feature. For example, if a query contains the words, “the Image object added to the addImage method,” then an IR model attempts to locate source code elements which contain these words (“added”, “Image”, “addImage” etc.). When a query and source code elements contain several shared words, the IR model gives the element a high relevancy score. Hence, IR-based FLTs accept, as input, a corpus of source code elements being analyzed, and queries. Execution of a query outputs a list of source code elements, ranked by the relevancy score between the input query and the elements.

**Fig. 1:**
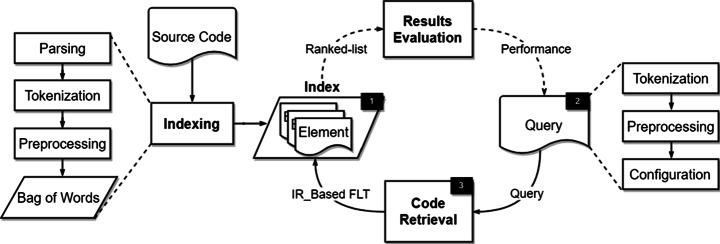
IR approach for feature location

Figure 1 depicts the overall IR approach for feature location, which also illustrates the evaluation procedure of FLTs. In general, the IR approach to the feature location problem can be described in three main steps where the evaluation procedure part loops over the latter two, as highlighted in the figure.

##### Indexing

The left side of Fig. 1 illustrates the source code indexing process. The process starts by extracting the textual content (e.g. comments and code identifiers) of source code elements (e.g. methods or classes). This is accomplished through lexical processing and parsing of the source code, producing a partitioned token stream. Then a series of transformations is applied to each token and produces one or more terms from each token. Common transformations include:

- Splitting code identifiers into their constituent words;
- Normalizing terms by converting them to a uniform case (upper or lower);
- Filtering terms by removing stop-words (e.g. ‘is’, ‘the’) and-or common programming language keywords (e.g. ‘if’, ‘else’);
- Stemming terms to reduce words to their inflectional roots (e.g. “protection”, “protective”, “protected” convert to “protect”).

The output of the process is usually the source code elements’ textual contents, in a compact matrix, where columns correspond to source code terms and rows correspond to the source code elements to which the terms belong.

##### Query Formation

Once a matrix/index is built, various FLTs can query it to locate features. The right side of Fig. 1 illustrates the source code analysis process in a loop. The process starts by formulating the queries from end-users or developers. These queries are typically pre-processed in the same manner as the source code: splitting, normalizing, filtering and stemming.

##### Code Retrieval

In the code retrieval step, after formulating a query, an FLT then applies the query to each source code element in the matrix. Finally, the FLT ranks the source code elements in descending order by their relevancy score to the query.

Result Evaluation refers to the evaluation of the FLTs’ results by a developer or by an FLT researcher, using the indexing, query-formation and code-retrieval steps of the approach. In FLT-researcher evaluation, multiple queries are formed, and run against the FLT technique, assessing the quality of the returned results against some known gold-standard result set or benchmark. For the past decade, researchers have sought to automate this task by employing the embedded text in software repositories as queries (Ali et al. 2013; Bassett and Kraft 2013; Binkley et al. 2015; Chochlov et al. 2017; Cleary et al. 2009; Rao and Kak 2011; Wang et al. 2011; Beard et al. 2011; Biggers et al. 2014; Eaddy et al. 2008; Mills et al. 2017; Thomas et al. 2013; Zhou et al. 2012; Dit et al. 2013; Moreno et al. 2015). For example, to formulate a query, researchers often use bug reports (the title, the description, or a combination of the two) from the issue tracking repositories of source code management systems. Then the ranked-list of elements returned by the FLT is compared against that gold-standard: for example, the locations of the fixes for the bugs in the repositories. Finally, a set of evaluation measures is employed to assess the comparison results using different aspects of effectiveness. In the real world scenario, the developer visually inspects the ranked-list of source code elements produced by the FLT for relevance.

#### Vector Space Model

The Vector Space Model (VSM) is a simple algebraic model. To demonstrate VSM, suppose *V* denotes the vocabulary in a set of source code elements *E*. Then an element *e* belonging to *E* is represented by a |*V* |-dimensional vector $\overrightarrow {W}$, where each entry of the vector represents the weight of a source code term belonging to *e* (Salton et al. 1975). Popular values for weight parameter are raw frequency (i.e. the number of occurrences of the term in *E*) or “tf-idf” (term frequency, inverse document frequency) (Mahmoud and Niu 2015; Thomas et al. 2013). Just like the elements of *E*, a query is also represented by a |*V* |-dimensional vector. The similarity between query and source code element is calculated by comparing their corresponding vectors. Popular similarity functions are “Euclidean distance,” “cosine distance,” “Hellinger distance,” or “KL divergence” (Thomas et al. 2013). Hence, VSM requires that queries should share the same terms with source code elements; the more shared terms they have, the higher their similarity score will be.

#### Latent Semantic Indexing

A source code element and a user generated query may use different terms when referring to the same feature. For example, a user may use synonymous (different terms used to describe the same concept) or polysemous (a single term having more than one distinct meaning depending on context) terms to describe the feature. In such cases, source code elements similar to a query will likely not be classified as such by VSM.

To address this issue, Latent Semantic Indexing (LSI), an extension to VSM, uses a “Singular Value Decomposition” (SVD) function to group the terms which are related by collocation (i.e., terms which often occur in the same elements). In this way, LSI projects the usage context of the terms in the form of “topics” prior to computing similarity. For example, a graphics-related topic might contain the words “image,” “colour,” “pixel” and “jpeg” because these words tend to appear in the same documents. In LSI, elements are still represented as vectors, but topic vectors, where LSI vectors contain the weight of topics. In contrast, VSM vectors contain the weights of single terms. To compare a user query, the query is first transformed into a topic space. Then, similarity between query and the elements in the topic space are measured by using the similarity function between their vectors (Deerwester et al. 1990).

In addition to the term weighting parameter and similarity function, LSI requires users to specify a “dimensionality-reduction” parameter which controls the number of topics (K) a user wants to populate during the SVD decomposition.

#### Latent Dirichlet Allocation

Latent Dirichlet allocation is a probabilistic topic model that provides a means to automatically index, search, and cluster unstructured documents. When used for code retrieval, similar to LSI, LDA uses the co-occurrence of terms in a corpus to discover the set of hidden topics (latent) within a corpus and represent each source code element as a finite mixture over this set of topics. In contrast to the LSI which uses SVD reduction to generate topics, in LDA topics are formed through an explicit generative process. This process usually employs Gibbs sampling (a machine learning algorithm) to iteratively assign words to the topics. Thus in LDA, each source code element is modelled given the probability that it expresses each topic, and each identified topic is modelled given the probability of a term from the corpus being assigned to the topic.

LDA calculates the probability that the source code element generates a term, given a set of terms belonging to a query *Q*. Thus the conditional probability (*P*) (i.e. similarity) of *Q* given a source code element *e* can be calculated as follows:

1 $$ Sim(Q,e)=P(Q|e)=\prod\limits_{qk \in Q} P(qk|e) $$

where *qk* is the *k*^*th*^ word in the query.

Other parameters to the LDA include: number of topics (*K*) to control the numbers of user-defined topics, the smoothing parameter for topic-to-element proportion (*α*) and the smoothing parameter for topic-to-term proportion (*β*). The latter two are hyperparameters which are used to tune the LDA model for a smoothing effect. In particular, a lower *α* value results in fewer topics per source code element and a lower *β* value results in fewer terms per topic, which generally increases the number of topics needed to describe a particular source code element (Lukins et al. 2008; Biggers et al. 2014; Corley et al. 2015).

### Issues in FLTs Evaluation

The presence of a large numbers of FLTs imposes difficulties for practitioners when deciding on the appropriate technique to employ for a given software maintenance task and for researchers when trying to identify the state-of-the-art techniques on which to build. This is due to two reasons:

1. FLTs evaluations are not fully characterized in terms of their FL goals (Razzaq et al. 2018; Shin et al. 2012) resulting in different, but implicit, evaluation biases being reflected in the researchers’ empirical design decisions;
2. The heterogeneity of empirical design is apparent from literature: Razzaq et al. (2018) suggested that over 60 different FLT evaluation metrics are used across the 170 papers with 272 subject systems having been used, and 235 different benchmarks employed. These heterogeneities make it very difficult to compare across FLT evaluations.

### Configuration of IR-Based FLTs and Best Practice

Given the same data-sets and evaluation measures, FLTs can still produce inconsistent or contradictory results because of the various configurations of FLTs and different empirical components like software system studied and queries generated (Thomas et al. 2013). In the IR models discussed above, for example, configurations depend on: weight of the terms in the index building step (e.g. frequency of the term in source code elements), number of topics used to reduce the terms into clusters and core similarity functions of a model (e.g. cosine similarity, Jaccard distance). It has been demonstrated in several studies that the performance of FLTs varies significantly by such configurations (Biggers et al. 2014; Thomas et al. 2013; Moreno et al. 2015; Panichella et al. 2016). Additionally, FLT empirical-settings, such as the way in which queries and source code are pre-processed, the entities of source code elements used for analysis (e.g. identifiers, comments, both) and how queries (e.g. bug report title, description, both) are formed for evaluation, have been shown to significantly impact the performance of FLTs (Biggers et al. 2014; Thomas et al. 2013; Moreno et al. 2015; Panichella et al. 2016). Hence, to perform an apple-to-apple comparison, consistent, and explicitly identified, FLT configurations and empirical settings must be applied towards a transparent evaluation.

There are several studies that investigate configuration and empirical settings in quantifying their impact on the performance of FLTs (Biggers et al. 2014; Thomas et al. 2013; Moreno et al. 2015; Panichella et al. 2016; Lukins et al. 2008). Commonalities between the findings of such studies and frequently employed settings by other empirical studies can be adopted towards a more homogeneous empirical design (Antoniol et al. 2002; Lukins et al. 2008; Marcus and Maletic 2003; Ali et al. 2013; Binkley et al. 2015; Cleary et al. 2009; Kagdi et al. 2013; Mahmoud and Bradshaw 2015; Poshyvanyk et al. 2006; Saha et al. 2013; Scanniello et al. 2015; Mahmoud and Niu 2015; Wang et al. 2011; Corley et al. 2015). Table 1 reports on these studies, and their identified best practices in FLT configuration and empirical settings. This paper, while assessing the relative effectiveness of each baseline technique, also employs these best-practice configurations and settings.

**Table 1:** Commonly employed configuration and normalization

Parameter	Total	Studies	Commonly
	Configurations	Empirically	Found
	Tested	Investigating	Best Practice
		Settings	
Settings Common to all FLTs			
Test Query	1. Bug report title	Thomas et al. (2013)	Title + Descr.
	2. Bug report description	Moreno et al. (2015)	
	3. Title + Descr.	Biggers et al. (2014)	
	4. Past 10 Bug Report		
	5. Past All Bug Report (PBR^*a*^-All)		
	6. Title + Descr.+PBR^*a*^-All		
Source Code	1. Identifiers	Thomas et al. (2013)	Ident. + Comm.
Entity	2. Comments	Moreno et al. (2015)	
	3. Ident. + Comm.	Biggers et al. (2014)	
	4. Literals		
	5. Ident. + literals		
	6. Comm.+ literals		
	7. Ident. + Comm.+ literals		
Pre-processing	1. None	Thomas et al. (2013)	Split+
Steps	2. Split	Moreno et al. (2015)	Stop+
	3. Stop	Panichella et al. (2016)	Stem
	4. Stem		
	5.Digit		
	6. Special chars		
	7. Split + Stop		
	8. Split + Stem		
	9. Stem + Stop		
	10. Split+ Stop+ Stem		
	11. Split+Stop+Stem+PBR^*a*^		
Specific to VSM			
Term Weight	1. Tf-Idf	Thomas et al. (2013)	Tf-Idf
	2. Sub-linear Tf-Idf	Moreno et al. (2015)	
	3. Boolean	Panichella et al. (2016)	
	4. tf	Wang et al. (2014)	
	5. tf-entropy		
	6. logij=log(tfij+ 1)		
Similarity	1. Cosine	Thomas et al. (2013)	Cosine
Function	2. Overlap	Moreno et al. (2015)	
	3. Jaccard	Panichella et al. (2016)	
	4. Dice	Wang et al. (2014)	
Specific to LSI			
Term Weight	1. Tf-Idf	Thomas et al. (2013)	Tf-Idf
	2. Sub-linear Tf-Idf	Moreno et al. (2015)	
	3. Boolean	Panichella et al. (2016)	
	4. tf		
	5. tf-entropy		
Similarity	1. Cosine	Thomas et al. (2013)	Cosine
Function	2. Jaccard	Moreno et al. (2015)	
	3. Dice	Panichella et al. (2016)	
Number of	32-300	Thomas et al. (2013)	200-300
Topics		Moreno et al. (2015)	
Specific to LDA			
Similarity	1. Conditional Probability	Thomas et al. (2013)	Conditional
Function	2. KL Divergence		Probability
*α*	0.01, 0.1, 0.25, 0.5, 0.75, 1	Biggers et al. (2014)	1
		Lukins et al. (2010)	
*β*	0.01, 0.1, 0.25, 0.5, 0.75, 1	Biggers et al. (2014)	Inversely
		Lukins et al. (2010)	proportional
			to the number
			of topics
Number of	32-500	Thomas et al. (2013)	200-300
Topics (K)		Biggers et al. (2014)	
		Lukins et al. (2010)	

^a^ Past Bug Reports

### Baseline Techniques: a Comparison Hub

This paper addresses the FLT comparability agenda, while also considering (implicit) FL goals, configuration parameters and design inconsistencies in previous studies. One solution towards addressing the issue is to employ a set of techniques that serve as common comparators across FLTs. Newly presented FLTs could then be compared to standard techniques that other researchers have also used to evaluate their FLTs. We argue that only by relative comparison of open, standard baseline techniques, under common evaluation measures, and other empirical-design conditions, will researchers begin to identify the high-performing FLTs in the field. Hence this paper empirically assesses such baseline techniques in empirical designs characterized by best identified empirical practice.

A baseline technique in this work is defined as a technique that serves as a common comparison vehicle: one that allows cross comparison between the FLTs. It is a standard technique, already proven as comparable, that can be repeatedly employed by researchers to facilitate subsequent comparisons across novel FLTs. Such a technique should be complete and openly available in a form that researchers can reproduce. It must be objective and not require programmer input into the FLT execution process.

**Fig. 2:**
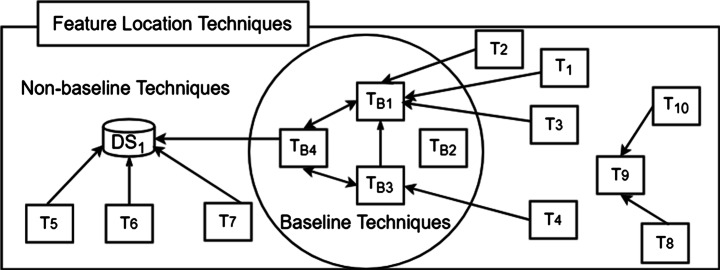
Scenarios of FLTs Comparisons and Demonstration of a Baseline Technique Facilitating Comparison Across FLTs

The comparison utility of baseline techniques is illustrated in Fig. 2 where T1 to T10 are some novel FLTs and TB1 to TB4 are baseline techniques. Each arrow in Fig. 2 represents an “empirically-compared-to” relation. Assuming relatively homogeneous empirical designs, T1 can be easily compared to T2 and T3 because they are all compared to the same baseline technique. Likewise, because TB3 has been compared to TB1, T1, T2, T3 and T4 can be indirectly compared, if that same homogeneity condition holds. On the right hand side, T5, T6 and T7 are techniques that were not compared with any baseline techniques, however they employed a shared dataset “DS1,” in evaluation, which has also been used to evaluate a baseline technique TB4. Suppose, the relative performance of TB4 with TB1 and TB3 is known for the same data-set, it allows an indirect comparison of T5, T6 and T7 with T1-T4. T8 and T10 are both compared to T9, where T9 is perhaps an FLT that does not have an openly-available executable. While this allows comparison of the two techniques, it does not allow their comparison to any of the other technique.

Razzaq et al. (2018) identified the following eight baseline techniques in a survey of FLT evaluations:

1. VSM-Lucene, Moreno et al. (2015)
2. VSM-Matlab, Dit et al. (2013)
3. VSM-Tracelab, Dit et al. (2015)
4. LSI-Gensim, Beard et al. (2011)
5. LSI-Matlab, Dit et al. (2013)
6. LDA-Gensim, Corley et al. (2015)
7. LDA-R, Biggers et al. (2014)
8. LDA-Gibbs, Zhou et al. (2012)

This paper assesses the relative performance of these baseline techniques towards allowing researchers to grade the performance of newly proposed FLTs that are compared to these techniques, against previously presented FLTs, that have been compared to these techniques. In addition, this will also facilitate backward comparability, facilitating cross-comparison of FLTs which have been evaluated against any one of the baseline techniques. In the next section we describe our empirical design to assess the relative performance of these baseline techniques.

## Empirical Design

This section presents the empirical design, in line with the guidelines provided in Wohlin et al. (2012), to evaluate software engineering techniques using case study research. The primary objective is to clearly present a context-based empirical frame-of-reference as suggested by Wohlin et al. (2012) (Section 3.1). The research questions targeted and hypotheses tested in this empirical assessment are described in Section 3.2. Software systems are selected as cases and the rationale for their selection is discussed in Section 3.3. Finally, the evaluation method, detailing the evaluation measures used and data collection and analysis methods employed to deal with each of the defined hypotheses, are described in Section 3.4.

### Empirical Frame-of-Reference

To classify the empirical design, we control for factors identified by Razzaq et. al. in FLT evaluations (Razzaq et al. 2018). Two major factors are described - goal-based classification of FLTs and evaluation criteria applied:

1. *Feature Location Goals* - The goals of FLTs are broadly classified into the following two categories (Razzaq et al. 2018):
  - *Near-full feature location*: Here the goal is to locate the full-extent of the source code implementing a feature (for example, identifying the full scope of a feature to be enhanced, or to define the differentz variants in a software system when moving to micro-services or a software product line Kästner et al. 2014). This goal has implications for estimation and resource allocation;
  - *Foothold-of-feature location*: Here the goal is to identify any single point-of-entry into the feature (a single source code element) as a “foothold”, for example to begin debugging or impact analysis (Poshyvanyk et al. 2012; Rovegård et al. 2008).
2. *FLTs Evaluation Criteria*: FLTs have been assessed using various objectives. However, effectiveness is the predominant objective, as suggested by Shin et al. (2012) and Dit et al. (2013) and Razzaq et al. (2018). Based on the specific evaluation goals of FLTs, effectiveness can be measured by two aspects (Shin et al. 2012):
  - *Relevance*: Precisely separating feature-related code from non-related code.
  - *Rank*: Ranking of the retrieved feature-related source code elements on the ranked-list.

Importantly, assessing an FLT with both of these effectiveness aspects combined is also a common practice in evaluation (Cleary et al. 2009; Mahmoud and Niu 2015; Zamani et al. 2014). Therefore, we will assess FLTs with respect to both effectiveness-based aspects and a composite (henceforth to be called relevance-rank criterion), using several commonly-employed metrics.

#### Research Objective

In this section, the defined empirical frame-of-reference is expanded to encompass the objective of this research. Our objective is to perform an exploratory assessment of baseline techniques for each goal of feature location with respect to the different effectiveness-based criteria of evaluation.

Specifically, the overall research objective can be divided into two:

1. *Near-full Objective*: Assess all baseline techniques for the near-full feature location goal.
2. *Foothold Objective*: Assess all baseline techniques for the foothold feature location goal.

With the near-full feature location goal, a user is normally interested in coverage analysis, where output of an FLT is assessed on the portion of the feature retrieved (Shin et al. 2012). Here an FLT can be assessed for both of the effectiveness-based criteria; i.e. how effective the FLT is by precisely separating feature relevant code from irrelevant code, and how effective it is in ranking the relevant source code elements highly on the retrieved list. Four evaluation measures were used to assess this (see Section 3.4.1).

An FLT that highly ranks at-least one feature related element is considered as more effective when the goal is to locate a foothold of a feature. Hence, to assess the baseline techniques with respect to the foothold location goal, we employed the effectiveness-based criteria of ranking relevant (potentially foothold) source code elements highly on the retrieved list, using MRR as our measure (See Section 3.4.2).

### Research Questions

We propose the following research questions to assess the baseline techniques:

- *RQ1.* Do the different baseline implementations of the same IR models perform differently for foothold and for near-full feature location goals?
- *RQ2.* What are the better baseline implementations of each IR model for foothold and for near-full feature location goals?
- *RQ3.* How do all the baseline techniques perform, relative to each other, for foothold and for near-full feature location goals? Which are the overall-best baseline techniques for both feature location goals?
- *RQ4.* Initial analysis of the results suggested that the performance of the FLTs depended on the individual characteristics of the systems/benchmarks to which they were applied. Hence a post-hoc research question assesses if differences in systems significantly impacted on FLT performance?

To further refine the research questions, we derive the null hypotheses to be tested in this empirical study. In order to represent these null hypotheses in a generic format, we employ the following nomenclature:

1. The IR-Models are referred to as IR-M_*X*_, where X can stand for VSM, LSI and LDA;
2. The three sets of Baseline Techniques, representing the baseline implementations of the VSM, LSI and LDA IR-Models respectively, are referred to as BT_VSM_, BT_*LSI*_ and BT_*LDA*_;
3. For each set of Baseline Techniques BT_*X*_, the individual elements in the sets are referred to as BT_*Xa*_, BT_*Xb*_...;
4. All the Baseline Techniques, regardless of the IR-M they implement, are referred to as BT_*a*_, BT_*b*_...;
5. The evaluation measures employed are referred to as E_1 − 5_;
6. The case studies are referred to as C_1 − 12_;

For *RQ*1, we tested whether the implementations belonging to an IR model (three in total), differs significantly from other implementations, of the same model, for each case study (twelve in total), for each evaluation measure (five in total). Thus one hundred and eighty hypotheses were tested in this regard. The following then, is the general null hypothesis that reflects *RQ*1:

For each IR-M_*X*_, for each case study C_1 − 12_ for each evaluation measure E_1 − 5_, each BT_*Xa*_ will perform similarly to all other BT_*X*_s;

That is, *RQ*1 assesses each implementation of an IR model for similarity. In contrast *RQ*2 assesses whether any implementation of an IR model significantly outperforms the other implementations of the same IR model and relates their magnitude of differences. Thus, implementations of an IR model are pairwise tested and related in terms of effect size for each of the twelve case studies employing all of the five evaluation measures. The following is the generalized null hypothesis:

For each pair of baseline techniques in BT_*X*_, for each case study C_1 − 12_, for each evaluation measure E_1 − 5_, BT_*Xa*_ will not significantly outperform BT_*Xb*_

Next, to measure the relative performance of all baseline techniques, we measure the difference in performance over all seven baseline techniques, for all four case studies. Thus, in answering *RQ*3, we pairwise-tested all of the baseline techniques for each case study and each evaluation measure. The following is the generalized null hypotheses:

For each pair of baseline techniques, for each case study C_1 − 12_, for each evaluation measure E_1 − 5_, BT_*a*_ will not significantly outperform BT_*b*_

In testing this category of hypotheses, and indeed *RQ*2, for each goal of FL, a baseline technique is considered to significantly outperform another if it is found to perform significantly better than the other baseline technique for the majority of cases.

Finally, to understand if software systems and their associated benchmarks impact on the relative performance of techniques, the following null hypotheses were retrospectively tested:

For each E_1 − 5_, if BT_*a*_ significantly outperforms BT_*b*_ on C_*X*_ then BT_*a*_ will significantly outperform BT_*b*_ on C_Y_

### Case Studies

Case studies, while providing in-depth insights, can make comparison across FLTs difficult. On the other hand, controlled experiments focus more on breadth of analysis, but might fail to control for the abundant extraneous variables that exist in the evaluation of FLTs (Razzaq et al. 2018; Ali et al. 2013; Bassett and Kraft 2013; Poshyvanyk et al. 2006; Wang and Lo 2014; Dit et al. 2013, 2011; Thomas et al. 2013; Shin et al. 2012; Panichella et al. 2016; Cataldo et al. 2009; Robillard 2008). They can also manifest as unreal, controlled situations which obscure the natural behaviour of FLTs (Easterbrook et al. 2008).

To simultaneously focus on depth (case-study) and width analysis, and to move towards a more standardized selection of the systems that provide trusted benchmarks, we adopted a multiple-case study design over carefully selected systems in this empirical assessment.

Case studies presented in FL literature range from very ambitious and well-organized studies in the field (Kagdi et al. 2013), to smaller, more toy-like examples (illustrations) (Chen and Rajlich 2000). Additionally, as suggested by Wohlin et al. (2012), the adaptable nature of “case study” research-design allows the accommodation of different research methods in it. To clarify, in the context of this study, we define the case-study context as:

“ an in-depth assessment performed on a non-sample software system. The employed software systems should be well-established systems, not just toy examples, where the features have their full (or nearly full) extent identified in the source code. This can be used as a benchmark in evaluating the location of features (Razzaq et al. 2018)”

In this section, we present the systems we studied and rationalize their selection. We required all selected systems to share the following characteristics:

1. *Open source*: To ensure the replicability of our case studies.
2. *Publicly accessible Issue Tracking System (ITS) and Version Control System (VCS)*: An ITS is a repository where developers (and users, in the case of publicly available systems) can report bugs or place feature-requests for a software system. When developers fix the filed bug or change features against a feature-request they update the status of the bug or feature-request in the ITS and store information on the source code elements updated during this process in a VCS. The process of reporting bugs or feature-requests in an ITS, and then acquiring the added, removed or changed source code elements, against them, from a VCS is known as re-enactment and, is one of the ground-truths (Li et al. 2013) used to create feature location benchmarks. In addition, we required the selected software systems to have ITS and VCS repositories publicly available to acquire the reported bug reports, feature-requests and code changes. During re-enactment bug reports/feature-requests can be used as a replacement for possibly subjective queries developed by researchers and the code changes, a proxy for feature locations (Mills et al. 2017).
3. *More reliable ground-truth*: Numerous benchmarks to validate the results of FLTs have been presented in FL literature. In previous work, we identified twenty-nine studies where they make their benchmarks publicly available (Razzaq et al. 2018). The majority of those benchmarks (twenty-four) are solely created through a single ground-truth. However, establishing a representative ground-truth is still a challenging and debatable subject (Ye et al. 2016; Tóth et al. 2010). To address this issue, researchers suggested either to employ multiple sources of ground-truths in the benchmark creation process (Hill et al. 2015) or select a set of source code elements which are commonly declared as benchmark elements by multiple researchers (Martinez et al. 2018). Hence, we required the benchmarks to be unambiguous; i.e. commonly accepted by researchers (Razzaq et al. 2018) or strengthened with triangulation: That is, there must be more than one ground-truth (Mills et al. 2017).
4. *Appropriate granularity*: Coarse-level (e.g. file-level) granularity of the benchmark elements requires additional effort by the developer to locate the exact feature-related elements (Chochlov et al. 2017; Kagdi et al. 2013), whereas ultra fine granularity (e.g. each line of a program) is unusual in studies. Therefore, for a software system to be selected as a case study in this empirical assessment, it must have a benchmark at method level (Eaddy et al. 2008) or one that can be easily converted into one at method level, where developers require less effort to locate the exact feature-related source code element (Chochlov et al. 2017).
5. *Different System types*: To address a typical concern with case-study research (generalizing its results) selected software systems should have diverse context parameters like project size, age of the system, application domain, type of issue tracking and version control systems. In this research, a system is considered to large, medium or small if that comprises-of more than 100K, 100-50K or less than 50K lines of code, respectively. We consider a system to be old if developed before 2010 and new if developed after 2010.

In addition to the above characteristics, we purposefully preferred to select software systems that have been employed in previous FLT evaluation studies to facilitate backward comparability of our results to the body of existing knowledge. We selected twelve software systems which meet the above criteria, from a list of 272 software systems applied in FLT evaluations (Razzaq et al. 2018). Table 2 presents the selected software systems and lists their diverse characteristics. Two of the systems are now discussed in fuller detail, to elaborate on types of ground-truths/benchmarks employed in this study.

**Table 2:** Characteristics of the selected case studies

	System	System	Features	Methods	Ground-	Issue Tracking
	Size	age			truth	System
ArgoSPL	Large	new	23	14,654	Conditional	Scarab
(family of versions)	($\sim $120KLOC)				compilation	
					directives	
Derby	Large	new	33	36,363	Re-enactment	Jira
10.9.1	($\sim $358KLOC)					
Eclipse	Large	old	45	123,732	Re-enactment	Bugzilla
3.3	($\sim $907KLOC)					
ArgoUML	Large	old	91	14,597	Re-enactment	Scarab
0.22	($\sim $149KLOC)					
jEdit	Large	new	150	6,413	Re-enactment	SourceForge
4.3	($\sim $99KLOC)					
Commons-Math	Large	new	63	14,845	Re-enactment	Jira
3.6.1	($\sim $85KLOC)					
muCommander	Large	old	92	8,187	Re-enactment	Trac
0.8.5	($\sim $77KLOC)					
JabRef	Large	old	39	4,607	Re-enactment	SourceForge
2.6	($\sim $74KLOC)					
Commons-Lang	Large	new	46	6,266	Re-enactment	Jira
3.5	($\sim $22KLOC)					
Rhino	Large	old	328	2,801	Prune	Bugzilla
1.5	($\sim $22KLOC)				dependency	
					+	
					Re-enactment	
					+	
					Document	
					mining	
iBatis	Large	old	85	1,869	Prune	Jira
2.3	($\sim $13KLOC)				dependency	
					+	
					Re-enactment	
					+	
					Document	
					mining	
Mylyn	Large	old	25	482	Prune	Bugzilla
1.0.1	($\sim $4KLOC)				dependency	
					+	
					Re-enactment	
					+	
					Document	
					mining	

#### ArgoSPL

ArgoUML^2^ is one of the most frequently studied software systems in feature location (Razzaq et al. 2018) and is extensively used by the extractive Software Product Line (SPL) adoption community (Assunção et al. 2017).

Martinez et al. (2018)^3^ have presented a feature location benchmark that uses the source code base of ArgoUML and ArgoUML variants. They leveraged the conditional compilation directives available in the ArgoUML SPL (Assunção et al. 2017) as an unambiguous and common ground-truth to generate this benchmark over the family of systems. These directives, like in C/C++, indicate to the pre-processor whether the delimited code fragment should be passed to the compiler or not. Hence, it enables full-extent definition of features and their cross-cuttings. Martinez et al. (2018) annotated these directives and provide a script to extract the respective source code implementations of 8 features and 15 cross-cutting features in ArgoUML. A cross-cutting feature is the set of source code elements shared by more than one feature. Hence, the feature set of the ArgoUML SPL comprises a collection of loosely coupled functional and cross-cutting features (Couto et al. 2011). The spread of a large code base over several cross-cutting features make it a challenging benchmark for feature location (Martinez et al. 2018).

Specifically, the benchmark contains a total of 23 feature to source code mappings corresponding to 8 loosely coupled features, 13 pair-wise feature cross-cuttings (the set of source code elements shared by two features), 1 three-wise feature cross-cutting (the set of source code elements shared by three features) and 1 feature negation (the set of source code elements not existing in the set of features). In these 23 feature-mappings, the benchmark has a total of 439 and 44 directly used and, 388 and 871 indirectly referred-to, classes and methods, respectively. To keep the granularity homogeneous in this assessment, we extracted all methods from the classes where the class was determined to be feature-related.

The list of features have already been described in the literature (Assunção et al. 2017). To eliminate any potential bias caused by queries formulated by users, these descriptions have been used as test queries (Martinez et al. 2018) in this research. To generate test queries for cross-cutting features, we aggregate the descriptions of the intersecting features.

#### Rhino

Rhino^4^ implements a formal specification: the ECMAScript Standard. Eaddy et al. (2008) reverse engineered the specifications of version 1.5R6 to derive its benchmark (feature to code mappings^5^). To verify the accuracy of the benchmark they employed a “prune dependency” rule. In prune dependency, a source code element is considered as related to a feature if it should be removed or altered, without affecting other features, when that feature is pruned. In this way, the prune dependency rule allows a direct partition of the software system into nuanced features, at the cost of intensive effort. To aid program understanding during the process of source-code-to-feature mapping using prune dependencies, Eaddy et al. (2008) relied on project documentation, source code comments, code navigation and search tools, change history comments, and unit tests.

For the purpose of formulating non-subjective test queries, Eaddy et al. (2008) also provide the feature-requests and bug reports assigned to source code elements using a re-enactment process. We employed their bugs-and-feature-requests mapping to further buttress/triangulate their benchmark: reverse engineering verified by prune dependency analysis, buttressed by re-enactment. This reduced the number of features when the associations disagreed.^6^ Finally, their presented benchmark was of relatively fine granularity (i.e. field and method level): To keep the evaluation design homogeneous in this study, we consider method level granularity only.

#### Other Systems

Other systems employed in this study are the commonly used systems in FLT evaluation literature (Eisenbarth et al. 2003; Bassett and Kraft 2013; Binkley et al. 2015; Mahmoud and Bradshaw 2015; Panichella et al. 2016; Thomas et al. 2013; Dit et al. 2015), for which a method-level benchmark exists, as suggested in the recent survey of the field (Razzaq et al. 2018). The benchmarks for these systems are created using a re-enactment process (Dit et al. 2013; Just et al. 2014), except for Mylyn and iBatis which, like the Rhino benchmark, used prune dependency and document mining (Hill et al. 2015; Eaddy et al. 2008).

The online repository presents the material^7^ used in our research, including feature-related benchmark elements and test queries gathered, as described above. Additionally, the online repository also includes the implementation of the baseline techniques, described in Section 2.3. Furthermore, it includes the intermediate results for all of the employed evaluation measures obtained in the assessment of the baseline techniques.

### Evaluation Method

We compared the results of baseline techniques by comparing the sorted (ranked) list of the source code elements obtained from them for the two FLT goals outlined. In this research, we employed the commonly used evaluation measures for each goal, as suggested by Razzaq et al. (2018).

#### Evaluation Measures: Near-full Feature Location

*Relevance Evaluation Criteria* - Evaluation of FLTs in the past have been conducted by measuring recall and precision on retrieved results (Antoniol et al. 2002; Marcus and Maletic 2003; Ali et al. 2013; Heck and Zaidman 2014; Mahmoud and Niu 2015; Gethers et al. 2011; De Lucia et al. 2011; Shin et al. 2012; Borg et al. 2014). Recall measures the accuracy of retrieved results whereas precision measures the extent to which only accurate results are retrieved.

Specifically, “recall” measures the fraction of feature-related source code elements that are correctly retrieved:

2 $$ \frac{|CorrectElements \cap RetrievedElements |}{|CorrectElements|} $$

“Precision” measures the fraction of retrieved source code elements that are related to the feature:

3 $$ \frac{|CorrectElements \cap RetrievedElements |}{|RetrievedElements|} $$

Precision alone fails to measure the coverage of the results, i.e. finding all of the feature-related elements, by ignoring not retrieved feature-related elements. Recall, by ignoring the incorrectly retrieved elements, fails to assess ranked-listing with lots of (distracting) false positives. Hence, to assess how precisely baseline techniques achieve nearly full FL with high accuracy, we deploy both of these measures.

Sometimes recall and precision are irreconcilable with respect to each other (Ali et al. 2013; Shin et al. 2012). “F-Measure” is another measure which gives a high value only in the case that both recall and precision values are high. It is a harmonic mean of recall and precision and is defined as follows:

4 $$ 2 \times \frac{|Recall \times Precision |}{|Recall + Precision|} $$

This paper employed all three measures, each having values in the range [0, 1].

Baseline techniques normally retrieve all of the source code elements of the software system in the ranked-list even if their relevancy to the test query is zero (Binkley et al. 2015; Zhou et al. 2012). In such cases, recall is always 100% and precision is always equal to the total feature-related elements over the total number of elements retrieved in the ranked-list (i.e. the total source code elements of the software system) (Poshyvanyk et al. 2007). Hence, precision and recall are not appropriate measures when assessed against the whole ranked list.

To address this issue, empirical studies have defined a threshold relevancy-score (Ali et al. 2013) or suggested selecting the top *K* elements (Antoniol et al. 2002) to measure precision and recall values. However, this practice still does not guarantee size-free assessment because such a threshold still favours or works against features having more or less feature-related source code elements than the threshold numbers, respectively (Shin et al. 2012). Additionally, variability in relevancy-score per technique and, even, per query could also be very high (Shin et al. 2012). Therefore, we used relative values instead of absolute values, as suggested by Shin et al. (2012), when evaluating near-full feature location. In doing so, for each feature, we measure the precision and recall values at the top 10 cut-points equal to size multiples of the benchmark elements related to that feature.

*Relevance-Rank Evaluation Criteria* - Mean Average Precision (MAP) is the most frequently used evaluation measure to assess the relevance-rank criteria in the FL literature (Shin et al. 2012; Razzaq et al. 2018). Average Precision(AP) measures the extent to which an FLT places correctly retrieved elements towards the top of the ranked-list by calculating the precision value at each position of the ranked-list where there is a relevant entry and then averaging those values. MAP, on the otherhand, measures the mean of the average precisions calculated for a set of queries. It can be represented as:

5 $$ \frac{1}{|Q|}\sum\limits_{q=1}^{Q}{\frac{{\sum}_{r=1}^{N}{(P(r) * isRelevant(r))}}{|RelevantElements_{q}|}} $$

where *r* is the rank position of a retrieved source code element in the ranked-list containing retrieved results of *N* such elements. Given a query *q*, *isRelevant*(*r*) is a binary function assigning 1 to the rank position *r* if it contains a feature-related source code element and 0 otherwise. *P*(*r*) is the function that computes precision after truncating the list immediately below the ranked position *r*. In this way, MAP assigns a higher precision score to feature-related elements at the top of the ranked-list (where the denominator is small) compared to the feature-related elements at the bottom (where the denominator is larger).

Note that “AP” is quite different from the “precision” measure. Precision is a single-value measure based on the whole list of source code elements returned by the technique. However, for a ranked sequence of source code elements, it is desirable to also consider the order in which the correctly returned elements are presented, which is what AP measures: a developer would typically have to scan source code elements in the list, presumably starting from the first one, until the relevant source code element is found. Since MAP measures the rank of accurate elements retrieved, it is used to simultaneously assess FLTs for their relevance-rank for the goal of near-full feature location in this study.

#### Evaluation Measures: Foothold Feature Location

The recall and the precision of results begin to be less important when the goal is to locate one feature-related element. Ranking measures that rank at the top of the ranked list becomes more influential, because they reduce unnecessary effort for the developer who vets the retrieval results. To perform this type of assessment, Poshyvanyk et al. (2007) initially defined an effectiveness metric that measures the rank of the first feature related element. This metric essentially represents the effort required by the developer in terms of the number of entities he would have to view before finding the first feature-related element (Poshyvanyk et al. 2012). However, the Mean Reciprocal Rank (MRR) is a more intuitive measure of higher ranks because it essentially puts the measure of Poshyvanyk et al. (2012) as the denominator, resulting in MRR being higher when the first feature-related element is at the top of the list and lower when the first relevant element is further down in the list. In addition, MRR is also a predominant choice in existing FLT evaluations when foothold location seems to be the goal (Lukins et al. 2010; Razzaq et al. 2018). While Reciprocal Rank(RR) measures the reciprocal of the rank position of the first feature-related element found in the ranked-list retrieved as a result of an FLT, MRR measures the mean of the reciprocal ranks over a set of test queries. MRR is defined as follows:

6 $$ \frac{1}{|Q|}\sum\limits_{q=1}^{Q}{\frac{1}{ranq_{q}}} $$

Where *rank*_*q*_ is the rank position of the top feature-related element calculated against a test query *q* from a set of queries *Q*. In this way, MRR assesses the FLTs for best case analysis in ranking one feature-related element (i.e. the foothold of a feature) towards the top of the ranked-list.

#### Data Collection Method

The overall data collection process is described as a formal procedure (see Algorithm 1). This facilitates a more effective automation process and reproducibility of our case study analysis for other researchers. The input to the procedure is a software system *S*, a technique to evaluate *T*, and certain configuration settings Ω for the technique *T*. The outputs produced by this process are the vectors representing the scores of each employed evaluation measure. The overall process, comprising of three phases, is discussed below. 
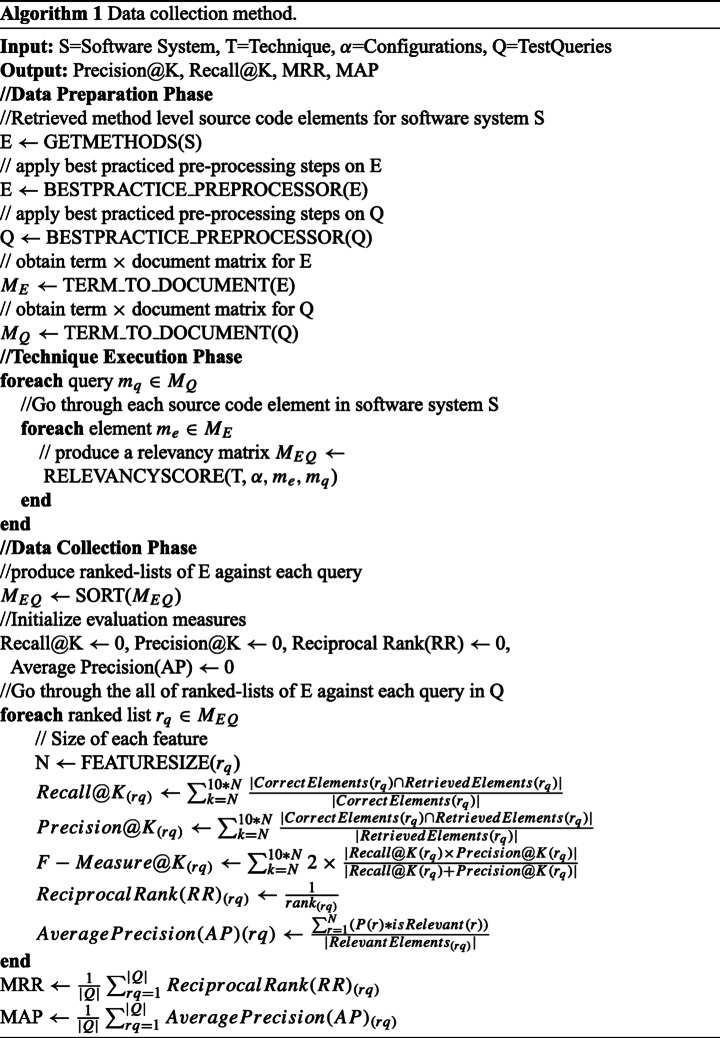


##### Data Preparation

The process is initiated by parsing the source code of each software system *S* at method level granularity, resulting in a list of all methods *E* from *S*. To accomplish this step, we applied a static program analysis to obtain the program model. We used the JDT API to obtain the Abstract Syntax Tree (AST) model of the program, and then extracted the source code elements (methods) from this AST model.

Next, the set of extracted methods *E* and the set of queries *Q* were pre-processed before execution of the FLTs. Considering the significance of empirical-settings in terms of its impact on FLTs performance, we applied the best identified empirical practice in this execution step, as discussed in Section 2.5. Specifically, identifiers and comments were both used to represent the source code elements whereas, title and description of feature-requests and bug reports are used to formulate the test queries. To pre-process the queries and corpus, all three major steps identified as having a positive impact on the FLTs performance; i.e. splitting identifiers, stop-word removal and stemming, were performed. In splitting the identifiers step, we combined camelCase and samurai (Dit et al. 2011) splitting techniques. For example, we split “myMethod,” “my_Method” and “my-method” into two isolated terms: “my” and “method.” For stop-word removal, we used the common English terms list provided by Dit et al. (2015) while presenting their reproducible framework for FLTs. In addition, we eliminate the non-literals and frequently used keywords (e.g. “if,” “else”) in the java language, in the spirit of Biggers et al. (2014) and Thomas et al. (2013). Finally, to stem the terms, we used the Porter stemmer (Porter 1980) implementation in apache Lucene.^8^

The pre-processed documents (queries and methods) are then converted to a standard representation used in IR-based FLTs (Rubin and Chechik 2013), i.e. a term × document matrix. Two such matrices, *M*_*E*_ and *M*_*Q*_ were created for each of the document sets *E* and *Q*, respectively. In *M*_*E*_, rows correspond to methods in *S* and columns correspond to the methods terms (i.e. the corpus vocabulary), whereas in *M*_*Q*_, rows correspond to test queries belonging to *S* and columns correspond to the query terms (i.e. the query’s vocabulary).

##### Technique Execution

In this phase, for each query *q* in the query set *Q* belonging to the software system *S*, we executed technique *T*, under configuration settings Ω, to find a relevancy score for all the source code elements *E* to *q*. To accomplish this, technique *T* was iteratively executed given each row of *M*_*Q*_ (corresponding to a query) to retrieve all rows of *M*_*E*_ which finally produced a relevancy matrix *M*_*EQ*_. In *M*_*EQ*_ each row corresponds to a query and each column corresponds to a source code element (method), whereas the value in each cell of *M*_*EQ*_ represents the relevancy score for each query to each element, according to the technique.

*RELEV* *ANCY* *SCORE*() is a general method passed with an instance of the baseline technique *T* and its configuration Ω, in addition to the source code elements and query. The baseline technique could be any of the eight techniques (i.e. VSM-Lucene, VSM-Matlab, VSM-Tracelab, LSI-Gensim, LSI-Matlab, LDA-Gensim, LDA-R and LDA-Gibbs) discussed in Section 2.3. In fact, all baseline techniques were employed in the studies. Ω is the frequently used configuration proven as best practice in producing better results as discussed in Section 2.5. Table 3 specifically lists the selected configuration for each of the baseline techniques and the examples of the studies which identified those configurations. Implementations of all baseline techniques are also provided in the replication package.^9^

**Table 3:** Configuration of the baseline techniques

Baseline technique	Optimal	Identified by
	Configuration	
VSM Lucene	TF-IDF,	Thomas et al. (2013), Moreno et al. (2015)
	Cosine Similarity	
VSM Matlab	TF-IDF,	Thomas et al. (2013), Moreno et al. (2015)
	Cosine Similarity	
VSM Tracelab	TF-IDF,	Thomas et al. (2013), Dit et al. (2013)
	Cosine Similarity	
LSI Gensim	Number of topics= 200	Marcus et al. (2004), Beard et al. (2011)
LSI Matlab	Complete	Dit et al. (2013)
	implementation as it	
LDA Gensim	number of topics: 200-500, *α*: 1,	Corley et al. (2015), Beard et al. (2011)
	iterations: 1000	
R LDA	number of topics: 200, *α*: 1,	Biggers et al. (2014), Lukins et al. (2010)
	iterations: 1000	
Gibbs LDA	number of topics: 200,	Zhou et al. (2012), Panichella et al. (2013)
	*α*: 0.5-1, *β*: 0.1,	

##### Data Collection

The final phase is the data collection phase where ranked lists of source code elements against each query are created. These ranked lists are then assessed with evaluation measures inferred by the assessment frame-of-reference of FLTs discussed in Section 3.1. The phase is initiated by relevancy score based sorting of source code elements in the *M*_*EQ*_ matrix, obtained in the technique execution step. Such a sorting creates a query-wise descending order sorting of source code elements in each row of *M*_*EQ*_, hence each row represents a ranked-list of source code elements retrieved against each test query. These ranked lists were then further assessed to calculate the values of the chosen evaluation measures. To measure the recall and precision at cut-points relative to the size of features under investigation, we determined the size of each feature *N*. Then the values of recall and precision were calculated at the first 10 cut-points equal to the 10 multiples of N. These recall and precision values were stored in vectors named “Recall@K”, “Precision@K” and “F-Measure@K” in algorithm 1, respectively. To measure the MRR and MAP, values of Reciprocal Rank(RR) and Average Precision(AP) were calculated for each ranked list *rq* and then averaged over the number of queries in *Q*. Finally, this process was repeated for all the instances of *S* (all software systems) and all instances of techniques *T* (all baseline techniques).

#### Statistical Analysis Method

To eliminate the risk of sample error, empirical assessment is undertaken on nearly complete sets of features in our selected case studies. However, to understand the underlying data distribution of selected feature sets responsible for such findings and to measure the statistical significance of our findings, (which potentially extends our work to other similar systems and new features in these systems in the future), we performed a statistical analysis on the results obtained.

Since, different implementations of the baseline techniques have been matched to each feature (each member of the data), we exploited paired statistic techniques to determine whether the performance difference of each baseline technique is statistically significant or occurred by chance alone.

In the case of normally distributed data, we exploited a paired t-test where there are two comparison groups and an “ANOVA” test where the comparison groups are more than two. Where the distribution of data was found to be non-normal, we leveraged the “Wilcoxon signed-rank” test in the case of two comparison groups and, in the case where there are greater than two comparison groups, the Friedman test was applied.

To verify the normality of the data, we used the “Shapiro-Wilk W” test which tests the null hypothesis, checking that the sample is drawn from a normally distributed population. To reject the null hypothesis in any of the selected paired tests, we accept a probability of 5% of committing a Type-I-error (i.e., *α* < 0.05). To measure the effect size, in order to find the magnitude of difference, for parametric analyses we used Cohen’s d. The effect size can be considered negligible for |*d*| < 0.2, small for 0.2 < |*d*| < 0.5, medium for 0.5 < |*d*| < 0.8, and large for |*d*| > 0.8. These thresholds are those suggested by Kampenes et al. (2007). Regarding non-parametric analyses, we employed the Cliff’s Delta (*δ*) effect size. We judge the magnitude of the effect size by comparing it to four thresholds suggested by Romano et al. (2006). These thresholds can be summarized as follows: negligible if |*δ*| < 0.147, small if 0.147 ≤ |*δ*| < 0.33, medium if 0.33 ≤ |*δ*| < 0.474, and large if |*δ*| ≥ 0.474.

Next we present the obtained results and discuss our findings in the context of each of the defined research questions.

## Results and Analysis

This section reports on the relative performance of baseline techniques in the evaluations. This includes results for and analysis of VSM, LSI and LDA baseline techniques for the first research question, gathered for each of the case studies, and these are presented in Section 4.1. For one large, medium and small system, we illustrate the results using boxplots, as they provide a quick visual representation of median, upper and lower quartile distributions, minimum and maximum values, and outliers. We further enhance the boxplots to present the mean value analysis. After demonstrating the performance of the implementations using box-plots, Sections 4.2, 4.3 and 4.4 present the results gathered for research question 2, research question 3 and research question 4, respectively. Finally, Section 4.5 summarizes the results and proposed answers to the research questions.

### Performance Variation of Different Implementations

#### VSM

##### Near-Full Feature location Goal

**Fig. 3:**
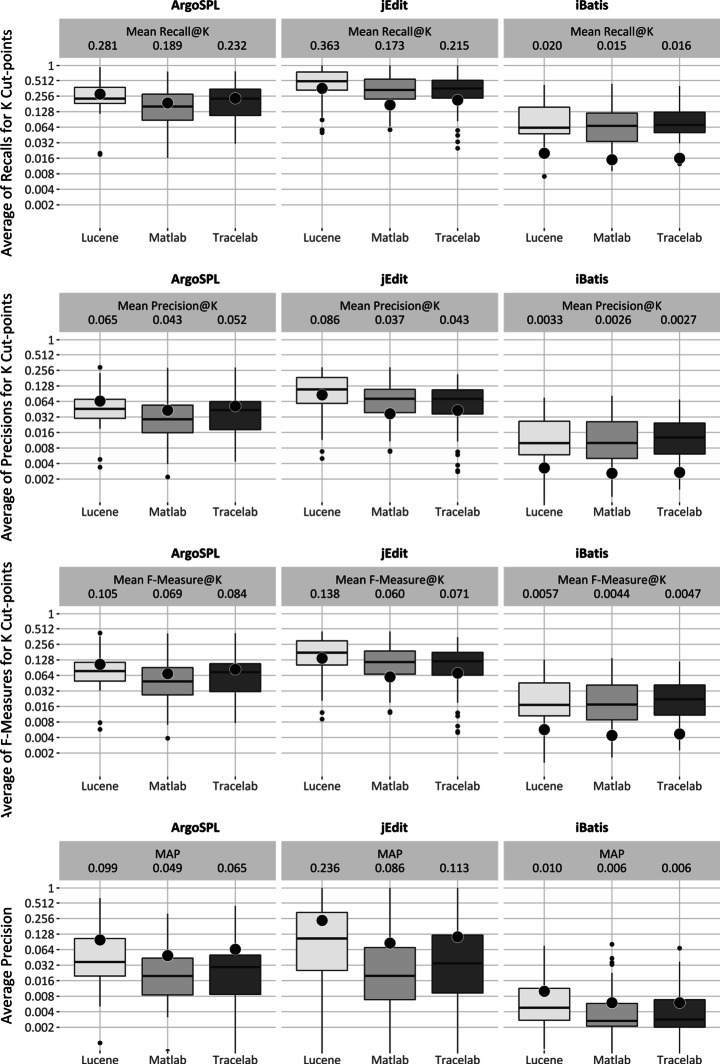
VSM performance for near-full goal of feature location

Figure 3 shows the recall, precision, F-measure and average precision for the Lucene, Matlab and Tracelab VSM implementations, applied to each of the three case studies (software systems) selected from different sizes. In the case of recall, precision and the f-measure, the boxplots in the figure plot the averaged values of the initial 10 cut-points. The full set of results for each cut-point are provided in our online repository. Dot points and horizontal lines through each boxplot represent the mean and median of the averaged values, respectively.

Even though the difference between systems dwarfs other differences, the difference in performance of VSM implementations is still visible from Fig. 3 on each system. The median, mean, and best performances differ for each implementation on each case study and the differences between the mean and median values are considerable. Their quartile distributions also differ significantly. As an example, consider the recall dispersion in ArgoSPL: the mean of the recall values averaged for the 10 cut-points (named Mean Recall@K) are 0.281, 0.189 and 0.232. The median values are 0.209, 0.128 and 0.172, and the best values are 0.933, 0.761 and 0.770 for Lucene, Matlab and Tracelab, respectively. This suggests that the choice of implementation for VSM model can have a large effect on the performance of a baseline technique.

##### Foothold Location Goal

**Fig. 4:**
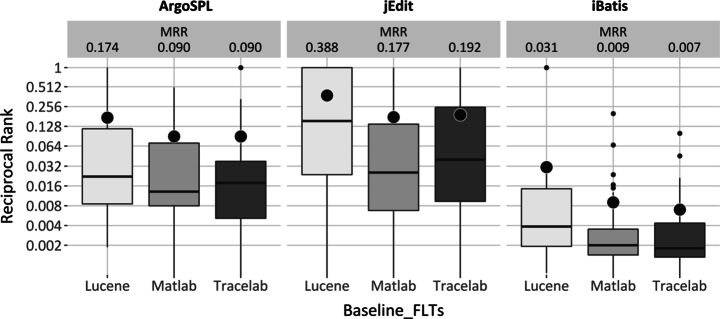
VSM performance for foothold location goal

Figure 4 displays MRR results for each case study. Clearly, the mean, median, best values, and distribution of results in the boxplot are quite different from one implementation to the others, even within systems. As an example, consider the differences for Lucene, Matlab and Tracelab VSM for ArgoSPL with respect to mean (0.174, 0.090 and 0.090), median (0.022, 0.013 and 0.018) and best values (1, 0.5 and 1).

#### LSI

##### Near-full Goal

**Fig. 5:**
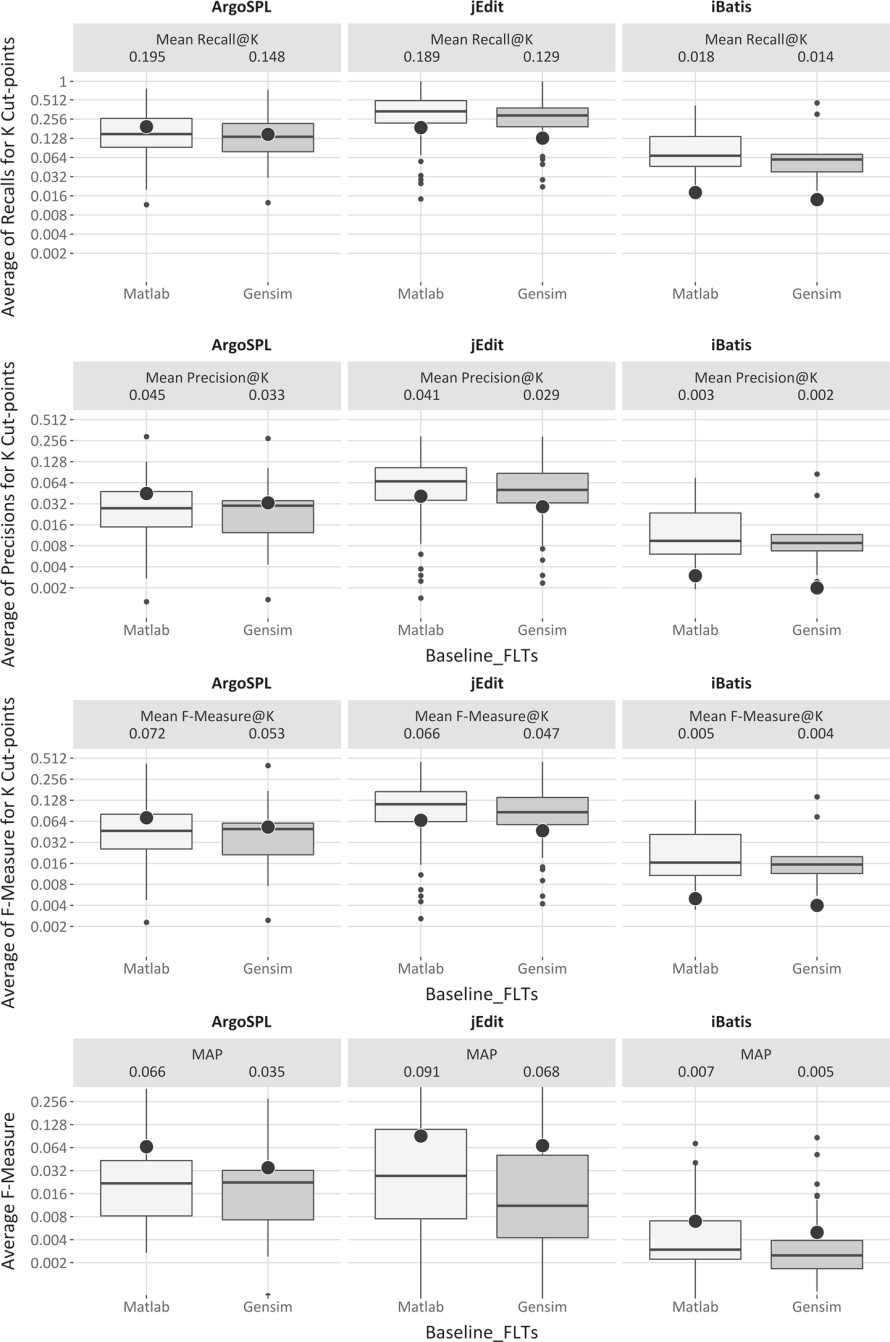
LSI performance for near-full goal of feature location

Figure 5 plots the recall, precision, f-measure and MAP results of two LSI-based implementations (i.e. Matlab and Gensim) over the same three systems. Again, it is evident that the results differ over LSI implementation, within each case studies. Consider iBatis for example: The Matlab implementation has higher quartile distributions than the Gensim implementation. Similarly, the mean and median values tend to be higher in the Matlab implementation, and these trends are visible across the three systems.

##### Foothold Location Goal

**Fig. 6:**
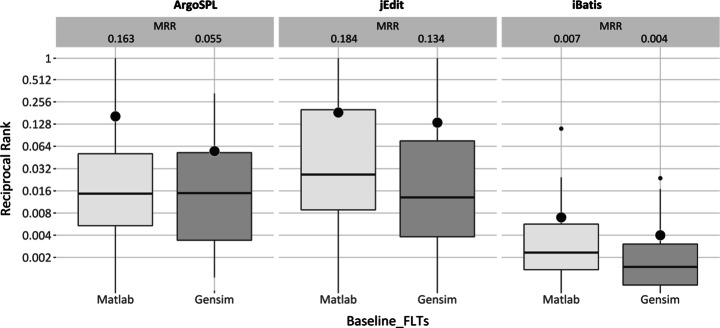
LSI performance for foothold location goal

Figure 6 visualizes the distribution of MRR results for Matlab and Gensim implementations of LSI. Regarding the quartile distributions, again there would seem to be a difference between the two: Matlab would seem to perform better than Gensim in all three case studies. In the case of median value analysis, Matlab performs at-least as well as Gensim, but usually better. In best value analysis, Matlab performs better or similar to Gensim for ArgoSPL and iBatis whereas in the case of jEdit, they perform similarly.

#### LDA

##### Near-full Goal

**Fig. 7:**
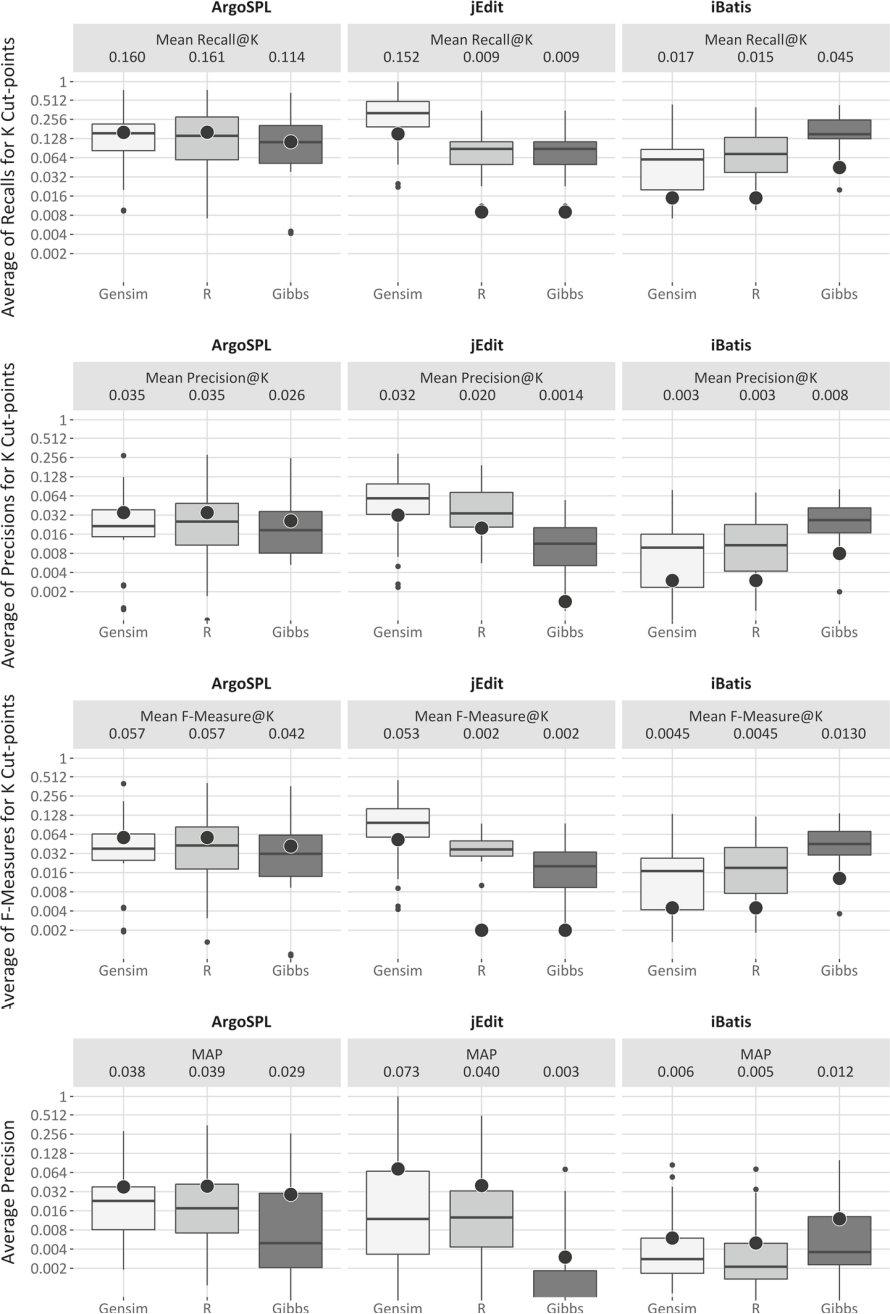
LDA performance for near-full relevance goal of feature location

Figure 7 displays the recall precision, f-measure and MAP dispersions of the three implementations of LDA for the three selected case studies. Consistent to the other IR models, LDA dispersions differ significantly across the case study systems, but in this case the differences seem inconsistent. Again differences across LDA implementations, but within systems, are also apparent from the box-plots.

##### Foothold Location Goal

**Fig. 8:**
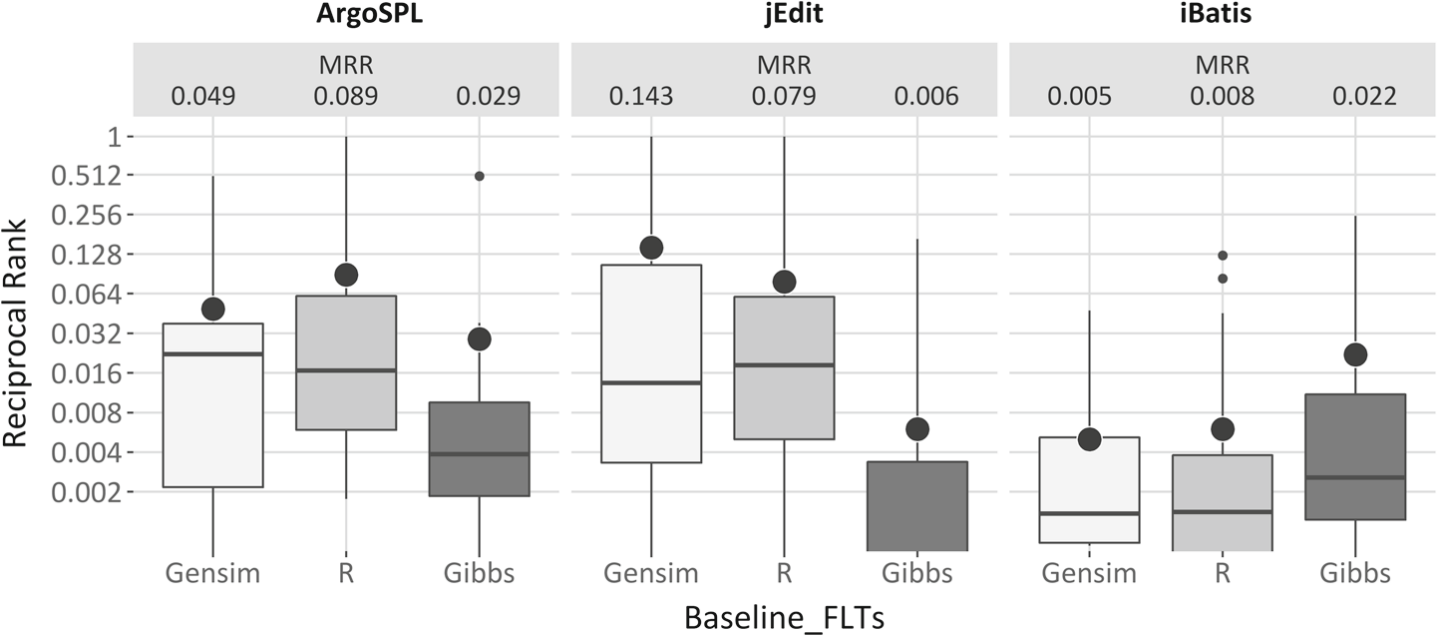
LDA performance for foothold location goal

Figure 8 displays MRR dispersion for Gensim, R and Gibbs. Similar to the other boxplots, the figure shows the different distributions across LDA techniques within systems, but that these differences are not always in the same direction across systems.

#### Differing Performance of Different Implementations

Table 4 presents the p-values for each of the hypotheses derived from research question 1 with respect to VSM, LSI and LDA.

**Table 4:** Performance difference of the different implementations belongs-to each IR-model

	Near-full Feature Location				Foothold Location
	Recall	Precision	F-Measure	MAP	MRR
VSM					
ArgoSPL	**0.001**	**0.014**	**0.014**	**0.003**	**0.019**
Derby	**0.000**	**0.001**	**0.000**	**0.000**	**0.000**
Eclipse	**0.000**	**0.000**	**0.000**	**0.000**	**0.000**
ArgoUML	**0.000**	**0.000**	**0.000**	**0.000**	**0.000**
jEdit	**0.000**	**0.000**	**0.000**	**0.000**	**0.000**
Math	0.071	0.439	**0.001**	**0.000**	**0.000**
muComman	**0.000**	**0.000**	**0.000**	**0.000**	**0.000**
JabRef	**0.002**	**0.001**	**0.001**	**0.000**	**0.000**
Lang	**0.000**	**0.000**	**0.000**	**0.000**	**0.000**
Rhino	**0.000**	**0.000**	**0.000**	**0.000**	**0.000**
iBatis	**0.006**	**0.010**	**0.010**	**0.000**	**0.000**
Mylyn	0.192	0.337	0.492	0.462	0.478
LSI					
ArgoSPL	**0.004**	**0.014**	**0.010**	**0.001**	0.715
Derby	**0.012**	**0.015**	**0.015**	**0.000**	**0.000**
Eclipse	0.655	0.655	0.655	**0.009**	**0.034**
ArgoUML	0.052	**0.042**	**0.038**	**0.002**	**0.005**
jEdit	**0.000**	**0.000**	**0.000**	**0.000**	**0.000**
Math	0.440	0.767	0.678	0.671	0.516
muComman	**0.000**	**0.000**	**0.000**	**0.000**	**0.000**
JabRef	**0.001**	**0.001**	**0.001**	**0.000**	**0.001**
Lang	0.055	0.040	0.509	0.091	0.073
Rhino	**0.000**	**0.000**	**0.000**	**0.000**	1.000
iBatis	0.211	0.156	0.156	**0.000**	**0.000**
Mylyn	0.300	0.233	0.211	0.326	0.412
LDA					
ArgoSPL	**0.006**	**0.014**	**0.014**	**0.000**	**0.000**
Derby	**0.001**	**0.000**	**0.001**	**0.000**	**0.000**
Eclipse	**0.044**	0.687	0.368	0.110	**0.028**
ArgoUML	**0.000**	**0.000**	**0.000**	**0.000**	**0.000**
jEdit	**0.000**	**0.000**	**0.000**	**0.000**	**0.000**
Math	**0.019**	**0.019**	**0.019**	**0.013**	**0.028**
muComman	**0.000**	**0.001**	**0.000**	**0.000**	**0.000**
JabRef	**0.001**	**0.001**	**0.001**	**0.000**	**0.000**
Lang	0.527	0.527	**0.044**	**0.000**	**0.000**
Rhino	**0.000**	**0.000**	**0.000**	**0.000**	**0.000**
iBatis	**0.001**	**0.000**	**0.000**	**0.000**	**0.000**
Mylyn	0.538	0.672	0.459	**0.009**	0.307

Table 4 is partitioned horizontally into three parts where each part presents the analysis results for each IR-model. Every row in the table presents the hypotheses results of a case study in terms of the p values for the performance difference assessed under *RQ*1, for all five evaluation measures. It can be seen from the p values in the rows that they are less than 0.05 (shown in bold) in the majority of the case studies for the majority of evaluation measures. This means that, we can successfully reject the null hypotheses that baseline techniques belonging to the same IR-model perform similarly for the majority of these evaluation measures in the majority of the case studies presented. Hence, the exact implementation of the IR technique should be referred to when reporting FLT evaluations. This finding is true for eleven of the twelve case studies, but implementations applied to Mylyn do not seem to differ significantly.

### Better-performing Implementations of each IR-model

Table 5 presents the hypotheses results for pair-wise analysis of the different implementations of the each IR-model, assessed under *RQ*2. Each row presents the effect-size for the hypotheses tested for all five evaluation measures. Arrow symbols in the table are used to indicate the increase or decrease of the first mentioned technique over the other. Embolding is used to demark significance. As an example consider the ArgoSPL system, and the embolded (0.656 *↑*) in the Lucene-over-Matlab row under the recall column. It indicates that Lucene performed significantly better than Matlab with an effect-size of 0.656 which is large according to Romano et al. (2006). Likewise, the “*↓*” symbol used in Matlab-over-Tracelab under the precision column indicates Tracelab performed better than Matlab with a medium effect-size.

**Table 5:** Pairwise performance difference of the baseline techniques belong to an IR-model^*a*^

System	Model	Implementation	Near-full Feature Location				Foothold Location
			Recall	Precision	F-Measure	MAP	MRR
ArgoSPL	**VSM**	**Lucene-over-Matlab**	**L(0.656)*↑***	**L(0.627)*↑***	**L(0.649)*↑***	**L(0.704)*↑***	S(0.26)
		**Lucene-over-Tracelab**	**L(0.615)*↑***	**L(0.545)*↑***	**L(0.553)*↑***	**L(0.66)*↑***	**L(0.498)*↑***
		**Matlab-over-Tracelab**	**M(0.428)*↓***	**M(0.389)*↓***	**M(0.381)*↓***	**M(0.425)*↑***	N(0.089)
	**LSI**	**Matlab-over-Gensim**	**L(0.607)*↑***	**L(0.514)*↑***	**L(0.537)*↑***	**L(0.679)*↑***	N(0.076)
	**LDA**	**Gensim-over-R**	N(0.054)	**N(0.031)*↑***	N(0.031)	N(0.025)	S(0.267)
		**Gensim-over-Gibbs**	**L(0.553)*↑***	**L(0.514)*↑***	**L(0.545)*↑***	**L(0.691)*↑***	**L(0.525)*↑***
		**R-over-Gibbs**	**L(0.504)*↑***	**L(0.478)*↑***	**L(0.478)*↑***	**L(0.622)*↑***	**L(0.694)*↑***
Derby	**VSM**	**Lucene-over-Matlab**	**L(0.641)*↑***	**L(0.641)*↑***	**L(0.641)*↑***	**L(0.804)*↑***	**L(0.801)*↑***
		**Lucene-over-Tracelab**	**L(0.557)*↑***	**L(0.55)*↑***	**L(0.55)*↑***	**L(0.732)*↑***	**L(0.664)*↑***
		**Matlab-over-Tracelab**	**S(0.147)*↓***	S(0.177)	S(0.177)	**M(0.44)*↓***	**M(0.425)*↓***
	**LSI**	**Matlab-over-Gensim**	**M(0.437)*↓***	**M(0.424)*↓***	**M(0.423)*↓***	**L(0.733)*↓***	**L(0.748)*↓***
	**LDA**	**Gensim-over-R**	**S(0.322)*↓***	S(0.279)	S(0.279)	**L(0.872)*↑***	**L(0.701)*↑***
		**Gensim-over-Gibbs**	**S(0.322)*↓***	S(0.279)	S(0.279)	**L(0.776)*↑***	**L(0.664)*↑***
		**R-over-Gibbs**	**S(0.322)*↓***	S(0.279)	S(0.279)	**L(0.627)*↑***	**L(0.872)*↓***
Eclipse	**VSM**	**Lucene-over-Matlab**	**M(0.405)*↑***	**M(0.381)*↑***	**M(0.376)*↑***	**L(0.937)*↑***	**L(0.96)*↑***
		**Lucene-over-Tracelab**	**M(0.405)*↑***	**M(0.378)*↑***	**M(0.376)*↑***	**L(0.969)*↑***	**L(0.979)*↑***
		**Matlab-over-Tracelab**	**M(0.405)*↑***	**M(0.378)*↑***	**M(0.376)*↑***	**L(0.937)*↑***	**L(0.96)*↑***
	**LSI**	**Matlab-over-Gensim**	N(0.067)	**N(0.067)*↑***	N(0.067)	**M(0.39)*↑***	**S(0.315)*↑***
	**LDA**	**Gensim-over-R**	S(0.25)	**N(0.146)*↑***	S(0.149)	N(0.066)	N(0.13)
		**Gensim-over-Gibbs**	**L(0.506)*↑***	**N(0.083)*↑***	S(0.149)	**S(0.184)*↓***	S(0.313)
		**R-over-Gibbs**	**S(0.313)*↑***	**S(0.188)*↓***	S(0.149)	N(0.016)	N(0.125)
ArgoUML	**VSM**	**Lucene-over-Matlab**	**L(0.5)*↑***	**L(0.505)*↑***	**L(0.502)*↑***	**L(0.644)*↑***	**L(0.535)*↑***
		**Lucene-over-Tracelab**	**L(0.512)*↑***	**L(0.505)*↑***	**L(0.509)*↑***	**L(0.634)*↑***	**M(0.452)*↑***
		**Matlab-over-Tracelab**	N(0.088)	N(0.109)	N(0.103)	**M(0.372)*↓***	N(0.139)
	**LSI**	**Matlab-over-Gensim**	S(0.204)	S(0.213)	S(0.217)	**S(0.317)*↑***	**S(0.298)*↑***
	**LDA**	**Gensim-over-R**	S(0.199)	S(0.169)	S(0.174)	N(0.07)	N(0.067)
		**Gensim-over-Gibbs**	**L(0.509)*↑***	**L(0.509)*↑***	**L(0.509)*↑***	**L(0.778)*↑***	**L(0.723)*↑***
		**R-over-Gibbs**	**L(0.568)*↑***	**L(0.568)*↑***	**L(0.568)*↑***	**L(0.779)*↑***	**L(0.748)*↑***
jEdit	**VSM**	**Lucene-over-Matlab**	**L(0.627)*↑***	**L(0.635)*↑***	**L(0.634)*↑***	**L(0.74)*↑***	**L(0.657)*↑***
		**Lucene-over-Tracelab**	**L(0.568)*↑***	**L(0.637)*↑***	**L(0.629)*↑***	**L(0.688)*↑***	**L(0.635)*↑***
		**Matlab-over-Tracelab**	**M(0.348)*↓***	**S(0.174)*↓***	**S(0.2)*↓***	**M(0.332)*↓***	**S(0.202)*↓***
	**LSI**	**Matlab-over-Gensim**	**M(0.402)*↑***	**S(0.318)*↑***	**S(0.327)*↑***	**L(0.475)*↑***	**M(0.343)*↑***
	**LDA**	**Gensim-over-R**	**L(0.542)*↑***	**S(0.173)*↑***	**L(0.554)*↑***	N(0.081)	N(0.022)
		**Gensim-over-Gibbs**	**L(0.542)*↑***	**L(0.541)*↑***	**L(0.541)*↑***	**L(0.76)*↑***	**L(0.696)*↑***
		**R-over-Gibbs**	N(0)	**L(0.502)*↑***	N(0.006)	**L(0.78)*↑***	**L(0.727)*↑***
Commons-Math	**VSM**	**Lucene-over-Matlab**	**M(0.344)*↑***	**S(0.208)*↑***	S(0.225)	S(0.222)	S(0.207)
		**Lucene-over-Tracelab**	**M(0.371)*↓***	**M(0.371)*↑***	**M(0.371)*↑***	**L(0.55)*↑***	**L(0.59)*↑***
		**Matlab-over-Tracelab**	**M(0.336)*↑***	**M(0.336)*↑***	**M(0.336)*↑***	**L(0.479)*↑***	**L(0.496)*↑***
	**LSI**	**Matlab-over-Gensim**	N(0.097)	N(0.037)	N(0.052)	N(0.053)	N(0.082)
	**LDA**	**Gensim-over-R**	S(0.278)	**S(0.278)*↑***	**S(0.278)*↑***	S(0.177)	S(0.169)
		**Gensim-over-Gibbs**	**S(0.278)*↑***	**S(0.278)*↑***	**S(0.278)*↑***	**M(0.379)*↑***	**M(0.389)*↑***
		**R-over-Gibbs**	N(0.135)	N(0.135)	N(0.135)	**S(0.321)*↑***	**S(0.285)*↑***
muCommander	**VSM**	**Lucene-over-Matlab**	S(0.219)	N(0.099)	N(0.095)	N(0.143)	N(0.048)
		**Lucene-over-Tracelab**	**L(0.524)*↑***	**L(0.509)*↑***	**L(0.521)*↑***	**L(0.626)*↑***	**L(0.597)*↑***
		**Matlab-over-Tracelab**	**L(0.538)*↑***	**L(0.527)*↑***	**L(0.53)*↑***	**L(0.728)*↑***	**L(0.684)*↑***
	**LSI**	**Matlab-over-Gensim**	**L(0.491)*↑***	**M(0.38)*↑***	**L(0.552)*↑***	**L(0.585)*↑***	**L(0.494)*↑***
	**LDA**	**Gensim-over-R**	N(0.008)	N(0.004)	**M(0.42)*↓***	N(0.135)	N(0.09)
		**Gensim-over-Gibbs**	**L(0.521)*↑***	**L(0.831)*↑***	**M(0.447)*↑***	**L(0.765)*↑***	**L(0.724)*↑***
		**R-over-Gibbs**	**L(0.484)*↑***	**L(0.486)*↑***	**L(0.482)*↑***	**L(0.71)*↑***	**L(0.684)*↑***
JabRef	**VSM**	**Lucene-over-Matlab**	**L(0.528)*↑***	**L(0.575)*↑***	**L(0.568)*↑***	**L(0.699)*↑***	**L(0.637)*↑***
		**Lucene-over-Tracelab**	**M(0.436)*↑***	**M(0.445)*↑***	**M(0.448)*↑***	**L(0.699)*↑***	**L(0.702)*↑***
		**Matlab-over-Tracelab**	S(0.303)	**M(0.346)*↓***	**M(0.341)*↓***	**L(0.476)*↓***	**S(0.224)*↑***
	**LSI**	**Matlab-over-Gensim**	**L(0.543)*↑***	**L(0.538)*↑***	**L(0.543)*↑***	**L(0.615)*↑***	**L(0.52)*↑***
	**LDA**	**Gensim-over-R**	**L(0.543)*↑***	N(0.133)	N(0.133)	N(0.011)	N(0.025)
		**Gensim-over-Gibbs**	**L(0.547)*↑***	**L(0.561)*↑***	**L(0.561)*↑***	**L(0.784)*↑***	**L(0.717)*↑***
		**R-over-Gibbs**	**L(0.554)*↑***	**L(0.548)*↑***	**L(0.548)*↑***	**L(0.833)*↑***	**L(0.72)*↑***
Commons-Lang	**VSM**	**Lucene-over-Matlab**	**L(0.534)*↑***	**L(0.534)*↑***	**L(0.534)*↑***	**L(0.519)*↑***	**L(0.531)*↑***
		**Lucene-over-Tracelab**	**L(0.534)*↑***	**L(0.534)*↑***	**L(0.534)*↑***	**L(0.717)*↑***	**L(0.733)*↑***
		**Matlab-over-Tracelab**	N(0)	N(0)	N(0)	**L(0.648)*↑***	**L(0.653)*↑***
	**LSI**	**Matlab-over-Gensim**	S(0.283)	S(0.302)	N(0.097)	S(0.249)	S(0.264)
	**LDA**	**Gensim-over-R**	N(0.075)	N(0.12)	N(0.12)	S(0.286)	S(0.276)
		**Gensim-over-Gibbs**	**S(0.325)*↑***	**S(0.325)*↑***	**S(0.325)*↑***	**L(0.75)*↑***	**L(0.761)*↑***
		**R-over-Gibbs**	**S(0.298)*↑***	**S(0.298)*↑***	**S(0.298)*↑***	**L(0.499)*↑***	**L(0.495)*↑***
Rhino	**VSM**	**Lucene-over-Matlab**	**L(0.483)*↓***	**L(0.867)*↑***	**L(0.867)*↑***	**L(0.867)*↑***	N(0)
		**Lucene-over-Tracelab**	**S(0.253)*↓***	**L(0.867)*↑***	**L(0.867)*↑***	**L(0.867)*↑***	**L(0.994)*↑***
		**Matlab-over-Tracelab**	**L(0.729)*↑***	**M(0.467)*↑***	**L(0.754)*↑***	**L(0.867)*↓***	**L(0.994)*↑***
	**LSI**	**Matlab-over-Gensim**	**L(0.867)*↑***	**L(0.867)*↑***	**L(0.867)*↑***	**L(0.867)*↑***	N(0)
	**LDA**	**Gensim-over-R**	**L(0.497)*↑***	**L(0.867)*↓***	**L(0.859)*↑***	**L(0.861)*↓***	**L(0.851)*↓***
		**Gensim-over-Gibbs**	**L(0.491)*↑***	**L(0.867)*↓***	**L(0.864)*↑***	**L(0.867)*↓***	**L(0.851)*↓***
		**R-over-Gibbs**	**L(0.476)*↓***	**S(0.312)*↓***	**M(0.352)*↓***	**L(0.826)*↓***	N(0)
iBatis	**VSM**	**Lucene-over-Matlab**	**S(0.236)*↑***	**S(0.196)*↑***	S(0.207)	**L(0.663)*↑***	**L(0.629)*↑***
		**Lucene-over-Tracelab**	S(0.208)	**S(0.208)*↑***	S(0.208)	**L(0.764)*↑***	**L(0.689)*↑***
		**Matlab-over-Tracelab**	S(0.167)	**S(0.155)***$****↓***	S(0.155)	S(0.202)	**S(0.22)*↑***
	**LSI**	**Matlab-over-Gensim**	S(0.166)	**S(0.154)*↑***	S(0.154)	**L(0.495)*↑***	**L(0.482)*↑***
	**LDA**	**Gensim-over-R**	N(0.018)	**N(0.028)*↑***	N(0.028)	**L(0.537)*↑***	N(0.114)
		**Gensim-over-Gibbs**	**M(0.372)*↓***	**M(0.387)*↓***	**M(0.384)*↓***	**S(0.218)*↓***	**M(0.424)*↓***
		**R-over-Gibbs**	**M(0.406)*↓***	**M(0.422)*↓***	**M(0.416)*↓***	**L(0.663)*↓***	**L(0.597)*↓***
Mylyn	**VSM**	**Lucene-over-Matlab**	S(0.156)	N(0.147)	N(0.128)	S(0.304)	S(0.218)
		**Lucene-over-Tracelab**	S(0.148)	N(0.109)	N(0.126)	S(0.299)	S(0.186)
		**Matlab-over-Tracelab**	S(0.193)	S(0.148)	S(0.159)	S(0.159)	S(0.251)
	**LSI**	**Matlab-over-Gensim**	S(0.207)	S(0.239)	S(0.25)	S(0.196)	S(0.164)
	**LDA**	**Gensim-over-R**	N(0.068)	N(0.114)	N(0.114)	S(0.159)	N(0.046)
		**Gensim-over-Gibbs**	S(0.207)	S(0.222)	S(0.233)	M(0.39)	N(0.137)
		**R-over-Gibbs**	S(0.227)	S(0.204)	S(0.204)	**M(0.46)*↑***	S(0.186)

^a^ L=Large, M=Medium, S=Small, N=None

In the pair-wise analysis of the implementations belonging to VSM, in 138 out of 180 comparisons, the implementations differed significantly. Specifically, in 120 comparisons where Lucene compared with other implementations, 97 cases are significant. Of those cases, Lucene outperformed the others in 94 cases while the others out-performed Lucene in only three cases. Of the 44 cases where it differed significantly to Matlab it outperformed it 43 times and was outperformed by Matlab only once. When it was specifically compared to Tracelab it significantly outperformed it 50 times and was outperformed only twice. While these results also suggest an impact of system and measurement choice, overall the Lucene implementation of VSM is better performing than the other two implementations over the vast majority of these evaluations.

In the case of LSI, implementations differ for only 36 of the 60 cases where the Matlab implementation was found to be significantly better than Gensim 31 times and Gensim outperformed Matlab significantly only 5 times. While less consistent than VSM Lucene, the results suggest that the Matlab implementation performed significantly better more often than the Gensim implementation.

On pair-wise analysis of LDA implementations, it is evident from the table that the Gibbs performance was significantly different to the Gensim or R implementations in 96 out of the 120 hypotheses. Of those 96 hypotheses, Gibbs outperformed Gensim or R only 22 times, whereas in the other 74 cases Gibbs was outperformed by the others. So it seems, on average, to be slightly weaker than the other two LDA implementations. The performance of the other LDA-based techniques (Gensim and R) is mixed. For example, only 19 of the 60 cases comparing the performance of Gensi and R implementations differed significantly. Out of those 19, Gensim outperformed R in 14 cases. Thus, it is very hard to decide the best implementation of LDA for (or regardless of) a specific FL goal, with the likelihood being that its relative performance keeps varying in each case study. Similarly, the situation is also not very clear when compare VSM implementations other than Lucene (i.e. Matlab with Tracelab): in 41 of the 60 cases Matlab and Tracelab differ significantly. In 24 of these, Matlab outperformed Tracelab whereas in 17 Tracelab outperformed the Matlab implementation. In these cases, the results seem to be more system specific than anything else.

Finally, since FLTs should ultimately be evaluated by goal, it is interesting to analyse Table 5 by FL goals. In terms of near-full FL, Lucene outperforms the others 79/96 times and is only outperformed twice (precision and recall on Commons-Math and Rhino, respectively). With respect to gaining a foothold in the code to the feature Lucene outperforms the others 18/24 times and is never outperformed by the others. In most of these cases, Lucene outperformed the others with large to medium effect-sizes. This evidence suggests that, if you are looking at either of the two FL goals, you should look to VSM-Lucene as the best-of-breed baseline technique implementing the VSM model.

Considering the FL goal type differences for LSI, Matlab outperforms Gensim 25 out of 29 times in terms of near-full feature location and 6 out of 7 times in terms of foothold location goal. These are fairly consistent findings where Matlab and Gensim differ (although they only differ significantly in 60% of cases).

Results of the LDA-based techniques are mixed for both FL goals. Gensim and R differ only 17 times with respect to the near-full goal, where Gensim outperformed R for 13/17 times. In the case of foothold location Gensim and R differ only 2 times where Gensim outperformed R in one case and R outperformed Gensim in another. However, Gibbs performed less well in both goals: 20/77 times in near-full FL whereas 2/19 times in terms of foothold location goal.

### Relative Performance of Baseline Techniques

This section presents the relative performance differences between the baseline techniques. To find the performance differences, results of all baseline techniques in terms of mean values for each evaluation measure, are compared to find the percentage of increase or decrease with respect to the mean values of other baseline techniques.

Table 6,^10^ presents the percentages for all evaluation measures. The table should be read from left to right. For example, consider the precision row in the ArgoSPL study. It shows that VSM_Lucene performed 20.2% better than VSM_Tracelab, VSM_Tracelab performed 13.5% better than LSI_Matlab, and LSI_Matlab performed 4.3% better than VSM_Matlab. The former two are significant, as shown by the bolded text, but the latter is not. Note that the table shows significant difference only with respect to the subsequent technique. For example, in the recall row under iBatis,the difference between LDA_Gibbs and VSM_Lucene is significant, whereas all the remaining adjacent pairs are non-significant. It is evident from the table that VSM_Lucene outperformed the majority of the other techniques in nine of the twelve case studies. In Mylyn this trend was also evident but not to a significant degree, while in Derby (one of the nine) LDA_Gibbs outperforms VSM_Lucene for two of the five evaluation measures. Indeed, LDA_Gibbs outperformed VSM_Lucene for four evalution measures on iBatis also. LSI_Matlab seemed to performed better for both of the FL goals on muCommander and recall on Rhino. These relative performance percentages could plausibly be employed towards cross-comparison of FLTs when employing a homogeneous empirical design. For example, in the ArgoSPL study, VSM_Lucene performed 38% better than VSM_Matlab with respect to precision. Then, in a homogeneous empirical design for precision, if a novel FLT “A” performed 10% better than VSM_Lucene and another FLT “B” performed 5% better than VSM_Matlab, then “A” could be considered to perform approximately 43% better than “B”.

**Table 6:** Relative performance of each flt for each case study

ArgoSPL																						
Recall	**VsL**	**>**	**17.9%**	VsT	>	15.5%	LsM	>	3.3%	VsM	>	15.5%	LdR	>	0.5%	LdG	>	7.7%	LsG	>	22.2%	LdGi
Precision	**VsL**	**>**	**20.2%**	**VsT**	**>**	**13.5%**	LsM	>	4.3%	VsM	>	17.8%	LdR	>	0.2%	LdG	>	7.1%	LsG	**>**	**19.5%**	LdGi
F-Measure	**VsL**	**>**	**19.7%**	**VsT**	**>**	**14.2%**	**LsM**	**>**	**4.1%**	**VsM**	**>**	**17.7%**	LdR	>	0.1%	LdG	>	7.4%	LsG	**>**	**20.2%**	LdGi
MAP	**VsL**	**>**	**33.1%**	LsM	>	0.7%	**VsT**	**>**	**24.7%**	VsM	>	21.7%	LdR	>	1.6%	LdG	>	8.6%	LsG	**>**	**17.6%**	LdGi
MRR	VsL	>	6.5%	LsM	>	44.7%	VsT	>	0.2%	VsM	>	1.5%	LdR	>	37.5%	LsG	>	10.5%	**LdG**	**>**	**41.5%**	LdGi
Derby																						
Recall	**VsL**	**>**	**59.2%**	LdG	>	32.8%	LdR	>	6.5%	LsG	>	35.7%	VsT	>	15.0%	LdGi	>	79.3%	VsM	>	45.3%	LsM
Precision	LdGi	>	41.7%	**LdR**	**>**	**5.2%**	**VsL**	**>**	**70%**	LdG	>	19.6%	VsT	>	19.2%	**LsG**	**>**	**78.4%**	VsM	>	37.9%	LsM
F-Measure	**LdGi**	**>**	**40.6%**	VsL	>	0.8%	LdR	>	69.8%	LdG	>	19.8%	VsT	>	19.0%	**LsG**	**>**	**78.5%**	VsM	>	37.9%	LsM
MAP	**VsL**	**>**	**49.3%**	LsG	>	10%	**LdG**	**>**	**78.1%**	LdR	>	46.2%	VsT	>	61.0%	LdGi	>	44.7%	**VsM**	**>**	**14.7%**	LsM
MRR	**VsL**	**>**	**53.3%**	LsG	>	12.9%	**LdG**	**>**	**45.8%**	VsT	>	81.8%	**LdGi**	**>**	**72.6%**	LdR	>	55.8%	**VsM**	**>**	**42.4%**	LsM
Eclipse																						
Recall	**VsL**	**>**	**0.0%**	**VsM**	**>**	**0.0%**	**VsT**	**>**	**32.6%**	LdG	>	9.4%	**LdGi**	**>**	**9.7%**	LdR	>	94.2%	LsM	>	98.5%	LsG
Precision	**VsL**	**>**	**0.0%**	**VsM**	**>**	**0.0%**	VsT	>	46.3%	LdR	>	62.9%	**LdG**	**>**	**49.1%**	LdGi	>	68.6%	LsM	>	98.5%	LsG
F-Measure	**VsL**	**>**	**40.2%**	**VsM**	**>**	**67.8%**	VsT	>	53.8%	LsM	>	63.9%	LdG	>	69.2%	LsG	>	50%	**LdR**	**>**	**100%**	LdGi
MAP	**VsL**	**>**	**0.0%**	**VsM**	**>**	**0.0%**	**VsT**	**>**	**40.8%**	LdGi	>	4.9%	**LdR**	**>**	**41.4%**	**LdG**	**>**	**93.0%**	LsM	>	37.4%	LsG
MRR	**VsL**	**>**	**0.0%**	**VsM**	**>**	**0.0%**	**VsT**	**>**	**18.9%**	**LdR**	**>**	**22.7%**	**LdGi**	**>**	**12.2%**	LdG	>	75.8%	**LsM**	**>**	**57.7%**	LsG
ArgoUML																						
Recall	**VsL**	**>**	**39.0%**	VsT	>	5.1%	VsM	>	3.3%	LsM	>	8.2%	LdR	>	14.2%	LsG	>	22.3%	**LdG**	**>**	**99.2%**	LdGi
Precision	**VsL**	**>**	**43.4%**	VsT	>	4.2%	LsM	>	0.7%	VsM	>	8.9%	LdR	>	16.5%	**LsG**	**>**	**20.2%**	**LdG**	**>**	**99.5%**	LdGi
F-Measure	**VsL**	**>**	**42.5%**	VsT	>	4.9%	VsM	>	0.3%	LsM	>	9.6%	LdR	>	15.6%	LsG	>	20.8%	**LdG**	**>**	**99.5%**	LdGi
MAP	**VsL**	**>**	**59.0%**	**VsT**	**>**	**1.1%**	**LsM**	**>**	**2.4%**	**LdR**	**>**	**8.5%**	**VsM**	**>**	**12.0%**	LsG	>	21.9%	**LdG**	**>**	**95.4%**	LdGi
MRR	**VsL**	**>**	**43.6%**	**LsM**	**>**	**18.6%**	**LsG**	**>**	**11.9%**	VsT	>	3.6%	LdR	>	3.9%	**VsM**	**>**	**15.8%**	**LdG**	**>**	**97.8%**	LdGi
jEdit																						
Recall	**VsL**	**>**	**42.6%**	VsT	>	10.7%	LsM	>	8.5%	**VsM**	**>**	**12.3%**	LdG	>	14.0%	LsG	>	82.2%	**LdR**	**>**	**64.5%**	LdGi
Precision	**VsL**	**>**	**50.1%**	**VsT**	**>**	**5.6%**	**LsM**	**>**	**8.4%**	**VsM**	**>**	**12.6%**	LdG	>	9.6%	**LsG**	**>**	**32.8%**	LdR	>	92.9%	LdGi
F-Measure	**VsL**	**>**	**48.3%**	VsT	>	7.3%	LsM	>	8.4%	**VsM**	**>**	**12.6%**	LdG	>	10.4%	LsG	>	95.0%	**LdGi**	**>**	**15.8%**	LdR
MAP	**VsL**	**>**	**52.0%**	VsT	>	19.5%	LsM	>	5.6%	**VsM**	**>**	**14.8%**	LdG	>	6.7%	LsG	>	41.5%	**LdR**	**>**	**93.2%**	LdGi
MRR	**VsL**	**>**	**50.5%**	VsT	>	4.3%	LsM	>	3.7%	**VsM**	**>**	**19.4%**	LdG	>	6.1%	LsG	>	41.1%	**LdR**	**>**	**91.9%**	LdGi
Commons-Math																						
Recall	**VsL**	**>**	**18.2%**	LsM	>	15.8%	**LsG**	**>**	**13.4%**	**VsM**	**>**	**7.3%**	**LdG**	**>**	**82.1%**	LdR	>	80.7%	**LdGi**	**>**	**100%**	VsT
Precision	**VsM**	**>**	**58.1%**	VsL	>	25.6%	LsM	>	12.2%	LsG	>	24.6%	**LdG**	**>**	**87.7%**	LdR	>	76.5%	**LdGi**	**>**	**100%**	VsT
F-Measure	VsL	>	24.2%	LsM	>	13.4%	LsG	>	17.4%	VsM	>	6.6%	**LdG**	**>**	**86.8%**	LdR	>	77.2%	**LdGi**	**>**	**100%**	VsT
MAP	**VsL**	**>**	**49.8%**	LsG	>	5%	LsM	>	40.7%	**VsM**	**>**	**4.5%**	LdG	>	84.6%	**LdR**	**>**	**54.4%**	VsT	>	48.1%	LdGi
MRR	VsL	>	34.1%	LsG	>	6.9%	LsM	>	41.6%	**VsM**	**>**	**6.1%**	LdG	>	85.8%	**LdR**	**>**	**64.7%**	LdGi	>	7.1%	VsT
muCommander																						
Recall	**LsM**	**>**	**22.9%**	VsM	>	1.1%	VsL	>	23.4%	LdR	>	18.4%	LdG	>	40.3%	**LsG**	**>**	**83.3%**	**VsT**	**>**	**78.0%**	LdGi
Precision	**LsM**	**>**	**23.8%**	VsM	>	0.9%	VsL	>	17.5%	**LdR**	**>**	**46%**	**LsG**	**>**	**85.8%**	**VsT**	**>**	**67.3%**	**LdG**	**>**	**55.6%**	LdGi
F-Measure	**LsM**	**>**	**23.6%**	VsM	>	0.8%	VsL	>	18.8%	**LdR**	**>**	**91.2%**	LsG	>	11.5%	VsT	>	72.1%	**LdG**	**>**	44.0%	LdGi
MAP	**LdG**	**>**	**9.5%**	**LsM**	**>**	**22.2%**	VsM	>	9.0%	VsL	>	8.7%	LdR	>	52.4%	**LsG**	**>**	**80.9%**	**VsT**	**>**	**66.5%**	LdGi
MRR	**LsM**	**>**	**15.9%**	VsM	>	13.2%	LdR	>	11.6%	**VsL**	**>**	**40%**	LsG	>	35.7%	**LdG**	**>**	**51.8%**	**VsT**	**>**	**88.1%**	LdGi
JabRef																						
Recall	**VsL**	**>**	**30.1%**	VsT	>	11.4%	LsM	>	20.5%	LdG	>	10.8%	VsM	>	17%	LdR	>	28.5%	**LsG**	**>**	**81.1%**	LdGi
Precision	**VsL**	**>**	**30.1%**	VsT	>	18%	LsM	>	7.7%	LdG	>	24.3%	VsM	>	2.5%	LdR	>	34.8%	**LsG**	**>**	**81.4%**	LdGi
F-Measure	**VsL**	**>**	**29.9%**	VsT	>	17.1%	LsM	>	10.3%	LdG	>	21.5%	VsM	>	6.2%	LdR	>	33.4%	**LsG**	**>**	**81.2%**	LdGi
MAP	**VsL**	**>**	**55.6%**	**VsT**	**>**	**5.9%**	LdG	>	8.2%	**LsM**	**>**	**18.9%**	LdR	>	12.2%	**VsM**	**>**	**27.4%**	**LsG**	**>**	**88.0%**	LdGi
MRR	**VsL**	**>**	**52.7%**	LdG	>	16.8%	LsM	>	1.0%	VsM	>	2.2%	VsT	>	24.6%	LdR	>	11.3%	**LsG**	**>**	**88.8%**	LdGi
Commons-Lang																						
Recall	**VsL**	**>**	**49.6%**	**LdR**	**>**	**28.4%**	**LsM**	**>**	**18.1%**	LdG	>	26.6%	**VsM**	**>**	**100%**	VsT	>	0.0%	LsG	>	0.0%	LdGi
Precision	**VsL**	**>**	**31.5%**	LsM	>	28.6%	LsG	>	30%	LdG	>	32.7%	LdR	>	100%	VsM	>	0.0%	VsT	>	0.0%	LdGi
F-Measure	**VsL**	**>**	**50.6%**	LsG	>	27.8%	LsM	>	3.1%	LdG	>	29.4%	LdR	>	100%	**VsM**	**>**	**0.0%**	**VsT**	**>**	**0.0%**	LdGi
MAP	**VsL**	**>**	**64.3%**	LsM	>	17.9%	LsG	>	31.3%	LdG	>	33%	LdR	>	78.1%	VsM	>	43.2%	**LdGi**	**>**	**10.2%**	VsT
MRR	**VsL**	**>**	**48.7%**	LsM	>	17.3%	**LsG**	**>**	**35.6%**	LdG	>	31.2%	LdR	>	85.9%	VsM	>	43.3%	**LdGi**	**>**	**10.0%**	VsT
Rhino																						
Recall	**LsM**	**>**	**0.6%**	**VsM**	**>**	**0.6%**	LsG	>	0.0%	**LdGi**	**>**	**0.5%**	**LdR**	**>**	**0.8%**	**VsT**	**>**	**2.6%**	**LdG**	**>**	**0.8%**	VsL
Precision	**VsL**	**>**	**8.8%**	**LdG**	**>**	**5.2%**	**LsM**	**>**	**0.8%**	**VsM**	**>**	**0.1%**	**VsT**	**>**	**1.1%**	**LdGi**	**>**	**0.1%**	**LsG**	**>**	**0.3%**	LdR
F-Measure	**VsL**	**>**	**5.8%**	**LdG**	**>**	**2.6%**	**LsM**	**>**	**0.8%**	**VsM**	**>**	**0.6%**	**VsT**	**>**	**0.5%**	**LdGi**	**>**	**0.1%**	**LsG**	**>**	**0.3%**	LdR
MAP	**VsL**	**>**	**6.7%**	VsT	>	0.3%	**LdGi**	**>**	**1.7%**	**LsM**	**>**	**1.5%**	**VsM**	**>**	**0.8%**	**LdR**	**>**	**3.3%**	**LsG**	**>**	**0.3%**	LdG
MRR	VsL	>	0.0%	VsM	>	0.0%	LsM	>	0.0%	LsG	>	0.0%	LdR	>	0.0%	**LdGi**	**>**	**49.4%**	**VsT**	**>**	**28.1%**	LdG
iBatis																						
Recall	**LdGi**	**>**	**55.9%**	VsL	>	10.9%	LsM	>	7.1%	LdG	>	0.6%	VsT	>	5.2%	LdR	>	4.8%	VsM	>	8.4%	LsG
Precision	**LdGi**	**>**	**56.9%**	VsL	>	11.2%	LsM	>	7.5%	VsT	>	1.4%	LdR	>	0.8%	LdG	>	2.6%	VsM	>	15.3%	LsG
F-Measure	**LdGi**	**>**	**56.6%**	VsL	>	11.2%	LsM	>	7.5%	VsT	>	1.6%	LdG	>	0.6%	**LdR**	**>**	**3.7%**	VsM	>	13.9%	LsG
MAP	LdGi	>	18.7%	VsL	>	31.1%	LsM	>	6%	LdG	>	2.7%	VsM	>	1%	VsT	>	9.8%	LdR	>	4.4%	LsG
MRR	VsL	>	31.2%	**LdGi**	**>**	**57.8%**	**VsM**	**>**	**14.1%**	LdR	>	6.6%	**LsM**	**>**	**4.7%**	**VsT**	**>**	**35.1%**	LdG	>	20.7%	LsG
Mylyn																						
Recall	VsL	>	10.2%	VsT	>	0.6%	LsM	>	3.8%	LsG	>	1.7%	VsM	>	13%	LdG	>	6.3%	LdR	>	1.2%	LdGi
Precision	VsL	>	13.2%	LsM	>	0.8%	VsT	>	4.4%	VsM	>	5.2%	LsG	>	5.7%	LdG	>	14.5%	LdR	>	1.4%	LdGi
F-Measure	VsL	>	12.3%	LsM	>	0.5%	VsT	>	5.0%	VsM	>	3.7%	LsG	>	7.3%	LdG	>	13%	LdR	>	1.4%	LdGi
MAP	VsL	>	33.6%	VsT	>	0.9%	LsG	>	1%	**LdR**	**>**	**2.7%**	**LdGi**	**>**	**2.4%**	LsM	>	1.4%	VsM	>	11.6%	LdG
MRR	VsL	>	45.9%	LsG	>	4.7%	VsT	>	10.3%	VsM	>	3.3%	LdR	>	6.3%	LsM	>	9.5%	LdGi	>	2.4%	LdG

### Impact of Software System

Probably the most startling finding, across the result set of the research questions is the impact of the software systems under study. For example VSM_Lucene, while outperforming other baselines for the majority of the evaluation measures for 10 out of 12 systems, is outperformed by LSI_Matlab in mu_commander for each evaluation measure. Likewise, when applied to iBatis, it was outperformed by LDA_Gibbs in four of the evaluation measures (three significantly).

Another example concerns LSI_Mathlab. It performed relatively well across evaluation measures in 10 of the 12 system, ranking between 1.2 and 3.8 in terms of relative technique performance. However, in Derby it was the worst-performing technique across evaluation measures and in Eclipse its average technique ranking, across the evaluation measures was 7.4 (out of eight).

Likewise the box-plots derived from our results often suggested system-specific trends. For example Fig. 3 suggests that feature location using VSM IR techniques is generally easier on jEdit, slightly less easy on ArgoSPL and more difficult on iBatis, across evaluation measures. Finally, if looked at by system, Mylyn is the only system on which performance differences of baseline techniques are not statistically significant for nearly all of the evaluation measures emlpoyed (see Table 5).

Consequently, Table 7 re-presents the scores of evaluation measures, by software system. Scores are found to differ significantly across systems with a p-value < 0.050. Boldface values are the best values of each technique for each system using each evaluation measure. It is clearly visible from this table that the performance of the baseline techniques varies more from case study to case study than from technique to technique. For example, consider the recall scores. The difference of the performance for VSM_Lucene between Rhino and Eclipse is 0.751 whereas the difference between the best (LSI_Matlab) and worst FLT’s (VSM_Lucene) performance on Rhino, using the same measure is just 0.045. Similarly, the largest performance difference between two FLTs on a system (jEdit) is 0.352, whereas largest performance difference of an FLT (LSI_Matlab) between two systems is 0.796. These findings are mirrored throughout the table, implying that software systems (and their associated benchmarks) have characteristics that can more strongly impact on the performance of the FLTs than the FLTs themselves.

**Table 7:** Scores of evaluation measures on software systems for each FLTs

	Small Systems					Medium Systems				Large Systems		
	Rhino	Mylyn	iBatis	Lang	jEdit	JabRef	muCom.	Math	ArgoSPL	ArgoUML	Derby	Eclipse
Recall												
VSM_Lucene	0.751	**0.134**	**0.020**	**0.105**	**0.363**	**0.272**	0.137	**0.078**	**0.281**	**0.185**	**0.014**	0.000
VSM_Matlab	**0.791**	0.114	0.015	0.000	0.173	0.122	**0.138**	0.011	0.189	0.109	0.000	0.000
VSM_Tracelab	0.776	0.121	0.016	0.000	0.215	0.190	0.008	0.000	0.233	0.115	0.002	0.000
LSI_Matlab	**0.796**	**0.120**	**0.018**	0.013	**0.189**	**0.171**	**0.179**	**0.065**	**0.195**	**0.104**	0.000	0.000
LSI_Gensim	0.786	0.117	0.014	**0.053**	0.129	0.071	0.049	0.054	0.148	0.083	**0.004**	0.000
LDA_Gensim	0.756	**0.098**	0.017	**0.038**	**0.152**	**0.132**	**0.103**	**0.044**	0.160	0.064	**0.006**	0.000
LDA_R	0.782	0.094	0.015	0.033	0.009	0.098	0.103	0.008	**0.161**	**0.096**	0.004	0.000
LDA_Gibbs	**0.786**	0.093	**0.045**	0.000	0.009	0.014	0.002	0.002	0.114	0.001	0.002	0.000
Precision												
VSM_Lucene	**0.284**	**0.025**	0.003	**0.022**	**0.086**	**0.057**	0.029	0.020	**0.065**	**0.041**	0.000	0.010
VSM_Matlab	0.246	0.021	0.003	0.000	0.037	0.023	**0.030**	**0.048**	0.043	0.022	0.000	0.010
VSM_Tracelab	0.246	0.022	0.003	0.000	0.043	0.040	0.002	0.000	0.052	0.023	0.000	0.010
LSI_Matlab	**0.248**	**0.022**	**0.003**	**0.015**	**0.040**	**0.033**	**0.039**	**0.015**	**0.045**	**0.022**	0.000	0.000
LSI_Gensim	0.243	0.020	0.002	0.011	0.029	0.015	0.013	0.013	0.033	0.017	0.000	0.000
LDA_Gensim	**0.261**	**0.018**	0.003	**0.007**	**0.032**	**0.030**	0.001	**0.010**	**0.035**	0.013	0.000	0.002
LDA_R	0.242	0.016	0.003	0.005	0.020	0.022	**0.024**	0.001	0.035	**0.020**	0.000	**0.005**
LDA_Gibbs	0.243	0.016	**0.008**	0.000	0.001	0.003	0.000	0.000	0.026	0.000	0.000	0.001
F-Measure												
VSM_Lucene	**0.411**	**0.042**	**0.006**	**0.035**	**0.138**	**0.094**	**0.048**	**0.032**	**0.105**	**0.067**	0.000	0.000
VSM_Matlab	0.374	0.035	0.004	0.000	0.060	0.038	0.048	0.017	0.069	0.037	0.000	0.000
VSM_Tracelab	0.372	0.037	0.005	0.000	0.071	0.066	0.003	0.000	0.084	0.039	0.000	0.000
LSI_Matlab	**0.378**	**0.037**	**0.005**	0.013	**0.066**	**0.055**	**0.063**	**0.024**	**0.072**	**0.037**	0.000	0.000
LSI_Gensim	0.370	0.033	0.004	**0.017**	0.047	0.024	0.003	0.021	0.053	0.028	0.000	0.000
LDA_Gensim	**0.388**	**0.031**	0.005	**0.012**	**0.053**	**0.049**	0.001	**0.016**	**0.057**	0.022	0.000	0.000
LDA_R	0.369	0.027	0.005	0.009	0.002	0.036	**0.039**	0.002	0.057	**0.033**	0.000	0.000
LDA_Gibbs	0.370	0.027	**0.013**	0.000	0.002	0.005	0.000	0.000	0.042	0.000	0.000	0.000
MAP												
VSM_Lucene	**0.292**	**0.049**	**0.010**	**0.068**	**0.236**	**0.159**	0.060	**0.068**	**0.098**	**0.085**	**0.069**	0.031
VSM_Matlab	0.263	0.030	0.006	0.002	0.086	0.044	**0.066**	0.019	0.049	0.031	0.001	0.031
VSM_Tracelab	0.272	0.032	0.006	0.001	0.113	0.071	0.005	0.001	0.065	0.035	0.004	0.031
LSI_Matlab	**0.267**	0.030	**0.007**	**0.024**	**0.091**	**0.061**	**0.084**	0.033	**0.066**	**0.035**	0.001	**0.001**
LSI_Gensim	0.252	**0.032**	0.005	0.020	0.068	0.032	0.026	**0.034**	0.035	0.027	**0.035**	0.000
LDA_Gensim	0.251	0.026	0.006	**0.014**	**0.073**	**0.067**	**0.093**	**0.018**	0.038	0.021	**0.031**	0.010
LDA_R	0.261	**0.032**	0.005	0.009	0.040	0.050	0.055	0.003	**0.039**	**0.034**	0.007	0.017
LDA_Gibbs	**0.271**	0.031	**0.012**	0.001	0.003	0.004	0.002	0.001	0.029	0.001	0.001	**0.018**
MRR												
VSM_Lucene	**1.000**	**0.113**	**0.031**	**0.092**	**0.388**	**0.337**	0.107	**0.098**	**0.174**	**0.212**	**0.143**	0.016
VSM_Matlab	**1.000**	0.052	0.009	0.002	0.177	0.131	**0.139**	0.035	0.090	0.079	0.001	0.016
VSM_Tracelab	0.506	0.058	0.007	0.001	0.192	0.129	0.020	0.002	0.090	0.086	0.032	0.016
LSI_Matlab	**1.000**	0.047	**0.007**	**0.047**	**0.184**	**0.133**	**0.165**	0.060	**0.163**	**0.119**	0.000	**0.002**
LSI_Gensim	**1.000**	**0.061**	0.004	0.039	0.134	0.086	0.064	**0.065**	0.055	0.097	**0.067**	0.001
LDA_Gensim	0.364	0.042	0.005	**0.025**	**0.143**	**0.160**	0.041	**0.033**	0.049	0.067	**0.058**	0.009
LDA_R	**1.000**	**0.051**	0.008	0.017	0.079	0.097	**0.121**	0.005	**0.089**	**0.082**	0.002	**0.013**
LDA_Gibbs	**1.000**	0.043	**0.022**	0.001	0.006	0.010	0.002	0.002	0.029	0.001	0.006	0.010

Another important observation from Table 7 is that, in line with our observations from the boxplots, there seems to be a near-ranking across systems where FL seems to be easier on some systems than on others. For example, the evaluation scores for Rhino seem consistently higher than jEdit, which in turn seem consistently higher than Mylyn, iBatis and then Eclipse. As these variations patterns are similar across evaluation measure, it implies that only the characteristics of the software systems/benchmarks are causing these patterns.

### Proposed Answers to the Research Questions

#### RQ1

: Figures 3-8 and Table 4 suggest that different implementations of each IR model (VSM, LSI and LDA) performed differently and that these differences were substantial. Thus, FLTs compared with even identically-named techniques are non-comparable and, this suggests that the exact implementation of the employed baseline technique should be referred to while reporting FLT studies.

#### RQ2

: It is evident from Table 5 that, overall, VSM_Lucene tended to outperform other implementations of VSM. These results are less emphatic in the case of Commons-Math, muCommander, Rhino, and iBatis, particularly with respect to VSM_Matlab, and in Mylyn there are no significant differences. But overall, its probably fair to declare VSM_Lucene as the best performing technique for near-full and foothold feature location.

With respect to LSI, LSI_Matlab performs significantly better than LSI_Gensim for the near-full feature location goal for seven of the twelve systems (see Table 5). But this was not found for Commons-Math, Commons-Lang, iBatis, Mylyn or ArgoUML, even though the (non-significant) trend was in the same direction (favouring LSI_Matlab over LSI_Gensim). However, in the case of Derby, LSI_Gensim performed better than LSI_Matlab.

Table 5 presents a more mixed picture for the remaining techniques: VSM_Lucene does seem to outperform VSM_Matlab and VSM_Tracelab but no real distinction can be made between VSM_Matlab and VSM_Tracelab. Finally, with respect to LDA, we found that LDA implementations perform different to each other. However it is very difficult to declare an implementation better than the other considering their performance variations on different systems. Even the worst performing LDA_Gibbs was the best performing in the case of iBatis. As has been already suggested, all these findings are declared with the caveat that the software system has a strong impact on the performance of baseline techniques.

#### RQ3

: In a large majority of cases, VSM_Lucene was found to outperform all other baseline techniques for both goals of FL. Hence, it should probably be employed as a comparator when researchers compare their FLTs with one baseline technique. Finally, the relative rank of baseline techniques, presented in Table 6, can be employed towards the cross-comparison of FLTs using homogeneous empirical designs to this study.

#### RQ4

: Table 7 shows that, for these techniques and systems at least, performance of the FTLs varies more from system to system than from FLT to FLT. The table also shows that the systems often impacted on the performance of FLTs in a consistent manner. For example, it seems easier to FL in Rhino than Eclipse using IR techniques. This impact on the performance of the FLTs would seem to be caused by differences of the systems/benchmarks, as the impact direction is not aligned with different evaluation measures.

## Discussion and Cross-comparison of FLTs

This section discusses the results obtained in the study and the extent to which the software systems under study impact on the performance of the FLTs evaluated. This leads to a scoping of the results obtained here and this scoping is presented. Finally, a comparison framework for empirical evaluations of FLTs is discussed.

### Empirical Basis for FLTs Evaluation

#### Comparison across FLT Evaluations

The study shows that even identically named techniques, belonging to the same IR model, perform differently and often the differences are substantial. For example, consider the list of relative performances on the ArgoSPL case study, presented in Table 6: The differences between the best and worst performing implementations of LSI for recall, precision, f-measure, MAP and MRR are 27.0%, 29.4%, 29.3%, 57.3% and 83.9%, respectively. The differences between the implementations of other IR models is similarly diverse. It should be acknowledged however, that, in the case of topic models, this may be partially due to the effect of different configurations used to fine tune the different implementations of LSI and LDA (Lukins et al. 2008; Biggers et al. 2014; Thomas et al. 2013; Corley et al. 2015). But in the case of the algebraic model (VSM), all the implementations studied here combined TF-IDF with the cosine similarity function to build a VSM model. This shows that some differences (at least) are based on inherent factors in the implementation; for example differences between the internal algorithms used to build a term-to-frequency matrix. This observation is particularly important given the ranking of (the algebraic model) VSM_Lucene across implementations.

In summary, in order to cross-compare FLTs, IR models offer objective and reproducible solutions to the FL problem, in the form of baseline techniques. However, actual differences in the results of baseline technique implementations belonging to the same IR model illustrate the importance of having an exactly-the-same ’compare-to’ implementation for evaluations. Razzaq et al. (2018) showed that 43% of the FLTs they reviewed in their SLR of the field were compared using VSM, LSI or LDA, but were compared against different or not-specified implementations of these techniques. In combination with that paper, the findings presented here suggest that apparent comparability across the techniques in the field may be more limited than previously anticipated.

#### Reproducability of FLTs

Recently, reproducability has been one of the hotly debated issues in software engineering research (Collberg and Proebsting 2016; Shull et al. 2008; Dit et al. 2015; Thomas et al. 2013; Martinez et al. 2018; Juristo and Gómez 2012; Scanniello et al. 2015). The findings presented here also suggest that a holistic description of algorithms and partial disclosure of their important attributes might not be sufficient to reproduce an FLT. In addition, reproducibility by non-compilable source code alone has been questioned in the past, as such source code does not guarantee the ability to generate an identical FLT (Bassett and Kraft 2013; Binkley et al. 2015); a problem noted by Collberg and Proebsting (2016). Therefore, for an FLT to be counted as reproducible, we argue that the paper presenting it should make available and refer to the executable or compilable source code.

#### Performance of IR Models with respect to FL Goals

**Fig. 9:**
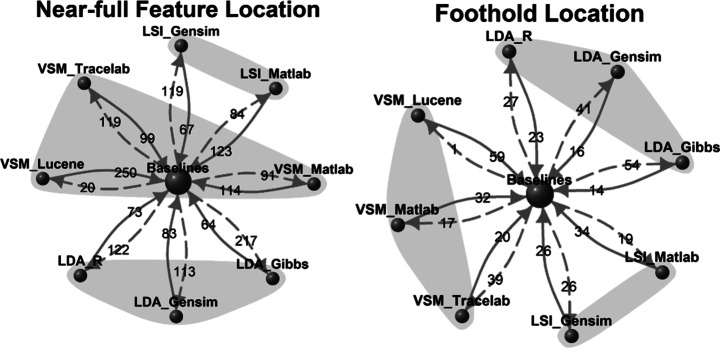
Number of times each baseline technique outperformed/being outperformed-by other baseline techniques for each FL goal

This study assessed baseline techniques using the most commonly employed evaluation measures in the field, for near-full and foothold FL. Figure 9 combines the results of the evaluation measures for each FL goal and presents an overview of the total number of times a baseline technique performed significantly better or worse than the remaining baseline techniques over the twelve systems. The line from a baseline technique to the “baselines vertex” in the centre of the figures, shows the number of times that technique performed significantly better than the other baseline techniques, whereas a dotted line from the “baselines vertex” to a baseline technique shows the number of times the other baseline techniques performed significantly better than the pointed-to baseline technique. It should be noted that the maximum number an edge could have is 336 for near-full feature location (four measures * twelve systems * seven compared-to techniques) and is 84 for foothold feature location (one measure * twelve systems * seven compare-to techniques). From this figure a ranked list of baseline techniques can be derived.

**Near-Full FL Goal**: VSM_Lucene > LSI_Matlab > VSM_Tracelab> VSM_Matlab > LDA_Gensim > LDA_R > LSI_Gensim > LDA_Gibbs

**Foothold Location Goal**: VSM_Lucene > VSM_Matlab > LSI_Matlab> LSI_Gensim > LDA_R > VSM_Tracelab > LDA_Gensim > LDA_Gibbs

Overall, the performance of algabraic models generally fares better than other IR models, particularly with respect to near-full feature location. This implies that the lexical space between feature queries and the implementation of the feature better encompasses the near-full FL problem than current term-proximities, or terms-to-topics probabilities, which are at the core of LSI and LDA (see Section 2.2), respectively.

In several earlier FL studies, where two or more FLTs were compared, it was suggested that VSM performs better than other IR models (Mahmoud and Niu 2015; Wang et al. 2011; Thomas et al. 2013; Zhou et al. 2012). However, other studies from the literature contradict this finding in favour of LSI (Antoniol et al. 2002; De Lucia et al. 2011). In the study presented here, both sets of findings are echoed: VSM_Lucene typically performs significantly better than LSI_Matlab but there are occasions (specific measures for specific systems) where the difference between the two is not significant and other occasions where LSI_Matlab significantly outperforms VSM_Lucene (see for example mu_commander in Table 6 where LSI_Matlab outperforms VSM_Lucene for every measure). Hence the contradictory results in previous studies are unsurprising, in that system heterogeneity has the potential to alter the rankings considerably.

There is one important note to be made regarding LDA for feature foothold location and LDA_R specifically: Its performance on feature foothold location is much better than on near-full FL. For example, for the systems presented in Fig. 8, its Mean Reciprocal Rank (MRR) generally lies above the one-third distribution of results, performing similarly to VSM and LSI (see Figs. 4 and 6). This shows that the LDA_R baseline technique normally locates one feature-related element in the upper quartile of the ranked-list positions, similar to the other FLTs. Interestingly, studies which used LDA_R for FL (Bassett and Kraft 2013; Binkley et al. 2015; Zhou et al. 2012; Corley et al. 2015) mostly employed MRR as their evaluation measure (Binkley et al. 2015), suggesting that knowledge of this alignment between LDA and the foothold feature location goal is already implicit amongst the community of researchers. But this is the first research that gives an explicit empirical basis to this implicit knowledge. Such goal-based differences in baseline techniques suggest that baseline techniques should always be separately assessed for foothold and near-full FL goals.

### Impact of Candidate Software System

The impact of the software system is one of the most startling, if post-hoc, findings of this study. For example, consider the performance of techniques for recall, precision, f-measure, MAP and MRR for small systems in Table 7: VSM_Lucene, LSI_Matlab, LSI_Gensim, LDA_Gensim and LDA_R generally performed better on Commons-Lang whereas VSM_Matlab, VSM_Tracelab and LDA_Gibbs generally performed better on iBatis. Considering the medium sized systems, VSM_Lucene, VSM_Tracelab, LSI_Gensim, LDA_Gensim and LDA_Gibbs performed better on JabRef, whereas VSM_Matlab, LSI_Matlab and LDA_R performed better on muCommander, for all of the evaluation measures. A similar pattern can be found in the Derby-Eclipse pair for large systems. These performance variations are mostly consistent across evaluation measures, which implies that there must be another factor at play. Given the consistent configurations of the FLTs and the homogeneous empirical design, the only independent variables which could have impacted on FLTs’ performance in such a way are the subject systems and their associated benchmarks.

An important observation from Table 7, is that the performance of all baseline techniques tend to vary in the same order across systems i.e. Rhino > ArgoSPL > jEdit > JabRef > muCommander > ArgoUML > Mylyn > Commons-Math > iBatis > Commons-Lang > Derby > Eclipse. In some cases this is very emphatic: every evaluation measure for every baseline technique scores better on Rhino than on ArgoSPL. In some cases it is less emphatic: techniques applied to ArgoSPL outperform those applied to jEdit in recall, precision and F-Measure for all but two baseline techniques, but this is reversed for MAP and MRR.

Several characteristics of these case studies might have impacted the baselines’ performance. For example, the table hints that size of the system may be a partial factor: Techniques applied to Eclipse and Derby, the two largest systems, performed worst, while techniques applied to the mid-sized systems seemed to rank in the middle. But techniques applied to ArgoSPL, another large system, performed 2nd best. Likewise of the small systems, techniques applied to Rhino performed best, but techniques applied to the other three small systems tended to be quite low down the rankings.

Another interesting, and possibly related, observation is that the performance of baseline techniques did not significantly differ in most cases for Mylyn. A possible reason may be that the Mylyn codebase is the smallest (482 methods) and the techniques differ more on larger systems. This suggestion is supported by the fact that iBatis and Commons-Lang, other smaller-sized systems, have a similarly tight distribution across results.

The average number of source code elements comprising a feature in the benchmarks might also have an effect on technique performance: the largest average number of source code elements per feature across our systems are 665 and 436 in Rhino and ArgoSPL respectively. Techniques applied to these two systems performed best and 2nd-best overall. Feature size probably reflects the number of meaningful lexicons that can be searched for in the code - a characteristic suggested by Chochlov et al. (2017) as an important indicator of IR technique effectiveness. However, feature-size by itself is probably not sufficient, given that other large features may have source code who’s meaningful lexicons overlap with the meaningful source code lexicons of the searched for features in highly coupled code.

More generally, in the vein of system characterization and FLT performance, we revisit the literature to look at system characteristics that have been shown to have some impact on FLT performance in the past:

1. Studies have shown that size of the software system (e.g. number of total source code elements) and complexity of its components can affect FLTs (Eaddy et al. 2008).
2. Coupling and cohesion between the elements related to the features have also been shown to have an impact on some FLTs (Wilde et al. 2001; Kagdi et al. 2013; Revelle et al. 2011).
3. The proportion of meaningful identifier names has been noted as increasing the performance of textual FLTs in several studies (Bassett and Kraft 2013; Dit et al. 2013).
4. Likewise, for a number of textual FLTs, the amount of comments has been shown to increase their performance (Chochlov et al. 2017).
5. Several characteristics of the software life cycle (e.g. code churn Thomas et al. 2013 or code ownership Diaz et al. 2013), have been found to impact on FLT performance (Kagdi et al. 2013; Diaz et al. 2013; Wang and Lo 2014; Ye et al. 2016; Thomas et al. 2013; Kim et al. 2013).
6. “Structures” are frequently used in the C language but rarely used in Java. Such differences in source code constructs between different languages have been shown to have some impact on the performance of FLTs (Saha et al. 2013; Wang et al. 2011).
7. Many FLTs leverage structural information (e.g. inheritance) to enhance FLTs (Bassett and Kraft 2013; Revelle et al. 2011; Saha et al. 2013; Wang and Lo 2014; Dit et al. 2013). Such program-structural information also differs across programming languages (Wilde et al. 2001; Revelle et al. 2011) and may impact the FLT.

This literature, and the findings from this study, suggest that looking more closely at software systems’/benchmark characteristics will be an important FL agenda in the future. Not only will it facilitate cross-comparison of FLTs, but it may also allow practitioners select the appropriate FLTs for their systems. To do so researchers will need to identify and evaluate system/benchmark characteristics that impact on FLTs and adopt empirical materials that test across the range of these characteristics.

### Forming a Common Baseline Assessment Matrix

This paper performed an assessment of the relative performance of baseline techniques towards cross-comparison of FLTs, as argued by Razzaq et al. (2018). Originally the aim was to find the best performing baseline FLT and relative rankings of baseline FLTs across systems and FL goals. But, given the huge variance of results across systems, this did not prove possible. Instead the core contribution of this paper is better contextualized as providing a comparison across baseline techniques, for each of a number of open system-benchmark pairs.

**Fig. 10:**
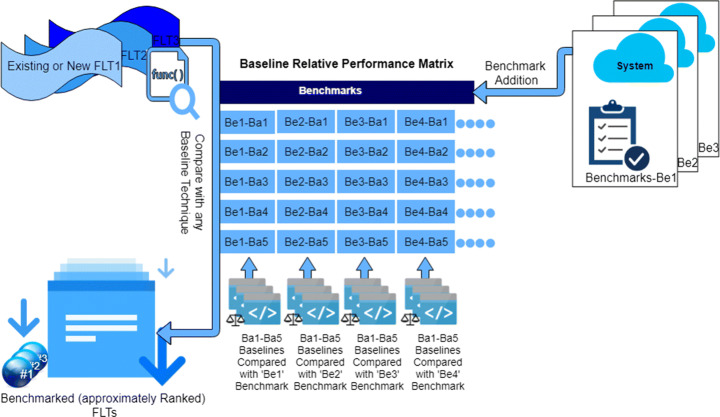
A framework to rank FLTs

Figure 10 presents the resulting framework. At the centre of the figure is the ”Baseline Relative Performance Matrix” where baseline techniques are compared against each other on the set of software systems utilized here, and their associated data sets (Fig. 10, right-hand-side). Given the baseline matrix, a novel FLT can be evaluated against these data-sets (Fig. 10, left-hand-side) using the empirical design espoused here and, by default, be assessed against all the baseline techniques currently in the matrix. Likewise all novel FLTs that are compared against this data set, using the empirical design espoused here, can be compared against each other, allowing researchers and practitioners a more holistic comparison across techniques, albeit on a limited selection of systems.

#### Forward Compatibility by the Framework

As time goes by, the research community can work to expand the matrix to address the limitation of the data-set. When more systems with high-quality benchmarks become available, researchers can evaluate the baseline techniques against the new systems, creating a new column in the matrix. Additionally, as new baseline techniques are provided, researchers can evaluate them against the systems already in the matrix, generating a new row in the matrix. And, as the matrix expands, it is envisaged that it will provide a rich environment for hypotheses regarding the interplay between system/benchmark characteristics and FLTs.

For example, an enlarged matrix can help to find sets of techniques (A) that perform better for sets of systems and sets of techniques (B) that perform better for other system. Then researchers can find how the characteristics of these sets-of-systems differ. This will ultimately help researchers to derive more nuanced benchmarks for FL and help move research towards ”FLT-recommender” systems based on existing system characteristics.

## Threats to Validity

### Construct Validity

#### Benchmark Creation

The main construct validity issue in FLT evaluation is the benchmark against which the FLTs are compared. In this case, the benchmarks had high-quality indicators: They were either commonly acccepted by the research community, provided by humans, system configuration directives or buttressed by triangulation/reverse engineering. But, even then, none of these practices guarantee an absolutely correct feature location set. Indeed, several expert developers highly familiar with the selected case studies, might each produce a different benchmark for the same feature. In addition, when certain types of dependencies are employed (e.g. prune dependencies) there is the possibility that other relevant dependencies (like database dependencies, XML file dependencies) are missed during the benchmark creation process. Likewise, information can be missing from re-enactment data-sets. For example, some of the links between bug-reports/feature requests and their fixing commits may be missing from CVS repositories. Given the size of the data-sets, we did not manually check that those links were complete.

#### Data Collection

The precision, recall and f-measure measures employed were based on the guidelines by Shin et al. (2012). However, selecting a percentage of elements, relative to the feature size is a heuristic approach that probably doesn’t reflect real-world practice. In addition, rank-based measures (e.g. MRR) only provide a proxy for effort in feature foothold location when, in reality, developers can often skim through the ranked-list. But these measures have frequently been adopted by previous studies in the field and are recommended in (Razzaq et al. 2018) for full feature location and feature foothold location respectively.

### Internal Validity

#### Query Designing

Involving actual developers in the query-building process might improve the quality of queries by leveraging the naming conventions they use when writing code, for example when naming variables, methods and classes. Although we employed external data-sets (benchmarks and queries) used by several studies (e.g. Poshyvanyk et al. 2012), bug-reports and feature-requests automatically extracted from online repositories may not accurately reflect developer queries when searching for a feature.

#### Configuration and Normalization

Configuration of the baseline techniques (weights, *α*, *β* and number of topics parameters) and data-set normalization options (e.g. stemming of source code and queries Binkley et al. 2015) could impact on the performance of FLTs. We employed the commonly-used, empirical-best-practice, verified for thousands of different settings, for each of the three baseline models, towards configuration of baseline techniques and normalization of the data-set (Biggers et al. 2014; Thomas et al. 2013; Moreno et al. 2015). However, as discussed earlier, individual FLT’s performance might also be impacted by other settings not yet fully explored, e.g. the size of queries and the size of the system. This will be a target of our future work.

#### Reproducibility

We used open implementations of the baseline techniques. However, these techniques comprise of multiple built-in functions which are combined by writing short scripts. A replicator may combine the functions in a different order that might impact on their reproducability. To address this issue, we provide the complete scripts used to implement the baseline techniques in this study. We further validated the reproducibility of baseline technique implementations by comparing the results with previous studies. For example, the MRR score of LSI_Matlab, for jEdit4.3, in Dit et al. (2013) was matched with the MRR score of the LSI_Matlab implementation used in this experiment, also on jEdit4.3. (Both were 0.18).

#### Result Calculations

The relative performance (percentage of the difference) between the baseline techniques is calculated after rounding the values to three decimal points for scores of evaluation measures. Relative performance calculated using different numbers of decimals might change the results slightly. Researchers interested in checking the results with a different number of decimals are invited to use the intermediate results provided with this research.

### External Validity

#### Selection of Software Systems

Case studies provide indepth insights, whereas controlled experiments tend to focus on generality (Easterbrook et al. 2008). To simultaneously focus on depth and generality analysis we adopted a multiple-case study design in this empirical assessment. To evaluate relative performance, all features (data points) from diverse case studies, are combined towards generalizing the findings, but ultimately only on open-sourced, java systems, developed using the Object-Oriented (OO) paradigm. This is by no means representative of all types of systems. More systems, taken from different domains, languages and paradigms would further improve the generality of this work, as would a greater number of Java, open-sourced and proprietary systems. This is particularly true given the diversity of results across systems noted in the results.

## Conclusion and Future Work

The vast numbers of FLTs proposed imposes difficulties for practitioners when deciding on the appropriate technique to employ for a given software maintenance task and for researchers when trying to identify the state-of-the-art techniques on which to build. We argue that only by relative comparison against open, standard baseline techniques, under common evaluation measures, and standard empirical-design conditions, will researchers begin to identify the high-performing FLTs in the field. In order to facilitate this comparison, this paper empirically assesses baseline FLT techniques against each other, using a set of high-quality system-benchmark pairs.

It formally defines an empirical design based on making explicit and standardizing several confounding factors. These include discriminating between different FLT goals and employing standardized evaluation measures against defined benchmarks for those goals. The FLT evaluation carried out employed the defined empirical design and performed twelve case studies to assess eight baseline techniques. The aim was to investigate whether different implementations belonging to baseline IR models perform differently and to identify the best implementation for each IR type. Later, in order to facilitate cross-comparison between FLTs, relative performance across all of the baseline techniques was also investigated. The following findings have been garnered from this work:

1. Different implementations of identically named baseline techniques perform differently in each empirical design, as characterized by different FL goals and evaluation criteria. This casts doubt on the cross-comparison of existing FLTs when compared with implementations of identically-named baseline techniques.
2. VSM-Lucene and LSI-Matlab are found to perform better than other implementations of VSM and LSI respectively, with the caveat that choice of software system may significantly impact their performance.
3. Overall, VSM-Lucene is found to be the best performing FLT for each FL goal in most of the case studies. Hence, we propose that VSM_Luecene be used as the default state-of-the-art baseline technique for comparison against newly proposed FLT techniques.
4. System effects dwarfed FLT effects in the results obtained. Additionally, there was a near-consistent ordering, across systems, in terms of the effectiveness of all the FLTs trialled, suggesting that some systems are more feature-location friendly than others, regardless of the approach employed. We hypothesize that this may be to do with feature/system size but that this needs further, directed exploration.
5. Rating factors, in percentages, that relate the performance of baseline techniques on the system-benchmark pairs were derived for the empirical design employed. These can be used to cross-compare the FLTs and ultimately provides a framework within which new and existing FLTs can be compared with less effort. Additionally this framework can be extended to new system-benchmark pairs, and provides the opportunity to gain insight into system characteristics that may impact on FLT performance.

We hope to extend this work to non-IR FLTs, by obtaining executable versions of several structural, dynamic and historical FLTs. These will then be assessed against the existing baseline techniques, using the system-benchmark pairs and the empirical design described here. In addition, we intend to keep searching for good candidate system-benchmark pairs that can be incorporated into, and expand, the framework.

Given the likelihood that different software system or features characteristics strongly impact on the performance of FLTs, we plan to target identifying these characteristics. This will facilitate selection of common system-benchmark pairs that not only provide trusted benchmarks, but also provide coverage over the relevant system characteristics, thus improving knowledge of the generality of the results obtained. Initial steps in this regard include studying the differing system characteristics in the Baseline Relative Performance Index, particularly for systems that have produced widely different results for the baseline techniques. Likewise, in expanding the set of baseline techniques assessed (to structural, dynamic and historical FLTs) this system-characteristics work can be extended to those techniques. Finally, in expanding the matrix with new system-benchmark pairs, the intention is to widen the data-set to a wider selection of system, ideally resulting in wider insights on FLT evaluation variations.

## Footnotes

1 https://www.lero.ie/research/datasets/feature_location/comparison

2 ArgoUML: http://argouml.tigris.org

3 https://github.com/but4reuse/argouml-spl-benchmark

4 http://www.mozilla.org/rhino

5 http://www.cs.columbia.edu/~eaddy/concerntagger/

6 This is why the number of features reported in the original study and the number we report are slightly different.

7 https://www.lero.ie/research/data-sets/feature_location/comparison

8 https://lucene.apache.org/core/7_2_0/analyzers-common/org/tartarus/snowball/ext/PorterStemmer.html

9 https://www.lero.ie/research/datasets/feature_location/comparison

10 VsL=VSM_Lucene, VsM=VSM_Matlab, VsT=VSM_Tracelab, LsM=LSI_Matlab, LsG=LSI_Gensim, LdG=LDA_Gensim, LdR=LDA_R, LdGi=LDA_Gibbs
